# Physicochemical and biological properties of three calcium silicate-based root canal sealers

**DOI:** 10.1371/journal.pone.0359096

**Published:** 2026-09-24

**Authors:** Xianyu Li, He Liu, Lari Häkkinen, Guoqiao Jiang, Binwen Chen, Markus Haapasalo, Ya Shen

**Affiliations:** 1 Department of Operative Dentistry and Endodontology, College of Stomatology, Hospital of Stomatology, Guangxi Medical University, Nanning, China; 2 Guangxi Health Commission Key Laboratory of Prevention and Treatment for Oral Infectious Diseases, Nanning, China; 3 Department of Oral Biological and Medical Sciences, Faculty of Dentistry, University of British Columbia, Vancouver, Canada; 4 Department of Stomatology, Tongji Hospital, Tongji Medical College, Huazhong University of Science and Technology, Wuhan, China; Universidade Federal Fluminense, BRAZIL

## Abstract

**Background:**

Calcium silicate based root canal sealers exhibit diverse physicochemical and biological properties that may influence periapical tissue responses. This study compared three sealers, Grey MTA Plus (MP), BioRoot RCS (BR), and Bio C Sealer (BC), using ProRoot MTA (PM) as a mechanistic reference material.

**Methods:**

Physicochemical properties, including pH, solubility in deionized water (n = 5), and elemental release (Ca, P, Bi, Pb, K, Mg; n = 3), were evaluated. Human periodontal ligament cells were used to assess cytotoxicity by CCK 8 assay, mineralization by Alizarin Red S staining, and gene and protein expression of IL 6 and MMP 1 by qRT PCR, ELISA, and Western blot (n = 3 independent experiments). MAPK pathway activation was analyzed in cells treated with BR. Statistical analysis was performed using one way ANOVA followed by Tukey’s post hoc test after verification of assumptions.

**Results:**

All materials exhibited alkaline pH values above 11. BC and BR showed higher solubility than PM under the tested conditions, whereas PM and MP released higher levels of calcium and were associated with increased mineralization. BC and BR reduced cell viability at higher concentrations and longer exposure times, particularly under serum free conditions. IL6 mRNA expression was reduced in BC and BR, but IL 6 protein levels were unchanged. In contrast, BR increased MMP 1 protein secretion. BR also induced phosphorylation of p38, JNK, and ERK1 2, and inhibition of these pathways reduced MMP 1 secretion.

**Conclusion:**

PM and MP showed higher calcium release and were associated with increased mineralization, whereas BC and BR showed higher solubility and concentration dependent cytotoxicity. BR increased MMP-1 secretion and was associated with MAPK activation. These findings are based on in vitro conditions and should be interpreted with caution.

## Introduction

The primary aim of root canal filling is to seal or entomb residual bacterial after chemomechanical disinfection and to support healing of periradicular tissues [1]. Root canal sealers are essential for filling irregular spaces within the root canal system and reducing leakage along the canal wall [2]. Because anatomical pathways such as the apical foramen, accessory canals, lateral canals, and dentinal tubules may allow sealer components to diffuse toward periapical tissues, the local tissue response to these materials is clinically relevant [3]. Therefore, both physicochemical behavior and biological effects should be considered when evaluating root canal sealers [4,5].

Calcium silicate based materials have attracted attention because of their alkalinity, calcium ion release, and potential bioactivity [6,7]. ProRoot MTA (PM; Dentsply Tulsa Dental Specialties, Tulsa, OK, USA) is a well characterized calcium silicate based material with favorable biocompatibility and bioactivity. However, it is not used clinically as a conventional root canal sealer because of its handling characteristics and prolonged setting time [8]. In the present study, PM was included as a mechanistic reference material rather than as a clinical sealer comparator. This reference provides a baseline for interpreting the behavior of newer calcium silicate based sealers derived from similar material chemistry [9].

Although the tested materials share calcium silicate chemistry, their formulations differ substantially. PM and Grey MTA Plus (MP; Avalon Biomed Inc., Bradenton, FL, USA) are powder and liquid systems mainly composed of tricalcium and dicalcium silicates, with bismuth oxide as a radiopacifier [10,11]. BioRoot RCS (BR; Septodont, Saint Maur des Fossés, France) contains tricalcium silicate, zirconium oxide, povidone, calcium chloride, and polycarboxylate, components that may influence setting, dissolution, and ion release. Bio C Sealer (BC; Angelus, Londrina, PR, Brazil) is a premixed sealer containing calcium silicates, zirconium oxide, and organic components such as polyethylene glycol to improve handling [12,13]. These compositional differences may affect solubility, alkalinity, ion release, cytocompatibility, and cellular responses.

The selected physicochemical and biological endpoints have direct relevance to root canal sealing and periapical healing. Solubility may affect the dimensional stability of the filling material and its ability to maintain a seal over time. Ion release, particularly calcium and phosphate release, contributes to alkalinity and apatite formation, which may be associated with mineral deposition and tissue repair [14,15]. However, excessive dissolution may also increase the concentration of released components and adversely affect surrounding cells. Cytotoxicity testing provides information on the tolerance of periapical cells to material leachates, while mineralization assays assess whether these materials support mineralized tissue formation. Inflammatory and matrix remodeling markers, including interleukin 6 (IL 6) and matrix metalloproteinase 1 (MMP 1), are relevant because periapical healing involves both regulation of inflammation and remodeling of the extracellular matrix [16,17].

Human periodontal ligament cells (hPDLCs) were selected as the biological model because they are among the principal cell populations located adjacent to the root apex. These cells may be exposed to sealer components when materials diffuse through dentinal tubules or are extruded beyond the apex. hPDLCs participate in inflammatory signaling, collagen turnover, extracellular matrix remodeling, and repair of periodontal and periapical tissues [10]. For these reasons, they provide a relevant in vitro model for examining cytocompatibility, mineralization, and matrix remodeling responses to root canal sealer leachates.

Despite increasing evidence on calcium silicate based sealers, the relationship between their physicochemical properties and cellular responses remains incompletely defined. In particular, it remains unclear whether differences in solubility and ion release are associated with changes in cell viability, mineral deposition, and expression of markers related to inflammation and matrix remodeling. The signaling pathways involved in sealer associated MMP 1 regulation also require further clarification.

The present study combined physicochemical testing with biological assays to evaluate material behavior from complementary perspectives. Solubility, pH, and elemental release were used to characterize material stability and ionic activity, whereas cytotoxicity, mineralization, gene expression, protein analysis, and MAPK pathway assessment were used to examine cellular responses. Together, these assays were intended to determine whether differences in material composition and physicochemical behavior are associated with distinct biological effects in hPDLCs.

Therefore, this study aimed to evaluate the physicochemical characteristics, including solubility, pH, and elemental release, and the biological effects, including cytotoxicity, mineralization, and markers related to inflammation and matrix remodeling, of three calcium silicate based root canal sealers, MP, BR, and BC, using PM as a mechanistic reference material.

## Materials and methods

This study was conducted in accordance with a protocol approved by the Clinical Research Ethics Committee of the University of British Columbia (Certificate No. H18-02897). Two maxillary first premolars were obtained from a healthy 26 year old woman undergoing extraction for orthodontic purposes. Written informed consent was obtained before tooth collection, and the patient was informed that the extracted teeth would be used for research.

The composition, lot numbers, and manufacturers of the tested materials are summarized in Table 1. All materials were prepared according to the manufacturers’ instructions.

**Table 1:** Composition, lot numbers, and manufacturers of tested materials.

Material	Composition	Lot number	Manufacturer
ProRoot MTA (PM)	Powder: tricalcium silicate, dicalcium silicate, tricalcium aluminate, tetracalcium aluminoferrite, calcium sulfate, bismuth oxide. Liquid: distilled water.	0000138162	Dentsply Tulsa Dental Specialties, Tulsa, OK, USA
Grey MTA Plus (MP)	Powder: tricalcium silicate, dicalcium silicate, tricalcium aluminate, calcium sulfate, gypsum, bismuth oxide. Liquid: water based gel with polymers.	2016030901	Avalon Biomed Inc., Bradenton, FL, USA
BioRoot RCS (BR)	Powder: tricalcium silicate, zirconium oxide, povidone. Liquid: calcium chloride, polycarboxylate.	03082720000	Septodont, Saint Maur des Fossés, France
Bio-C (BC)	Tricalcium silicate, dicalcium silicate, tricalcium aluminate, calcium oxide, zirconium oxide, silicon oxide, polyethylene glycol.	43919	Angelus, Londrina, PR, Brazil

This in vitro study evaluated four calcium silicate based materials: PM, MP, BR, and BC. The primary experimental factor was material type, with four levels. For cytotoxicity assays, leachate dilution and exposure time were also analyzed. Outcome variables included pH, solubility, elemental release, cell viability, mineralized nodule formation, gene expression, protein expression, and MAPK pathway activation.

All materials were prepared by a single trained operator to reduce procedural variability. When applicable, measurements and data analysis were performed by an investigator who was not involved in specimen preparation. Independent experiments were conducted on separate occasions using newly prepared material specimens or independently cultured cells.

Unless otherwise stated, *n* represents independent specimens for physicochemical tests or independent experimental runs for cell based assays. Technical replicates, including repeated measurements or replicate wells within the same independent experiment, were averaged before statistical analysis and were not treated as independent biological replicates.

### Physicochemical properties

#### Sample discs and leachate preparation.

Silicone molds measuring 9.5 mm in diameter and 1.5 mm in height were used to prepare discs of the tested materials, following a previously described protocol [18]. PM, MP, and BR were mixed manually on a sterile glass slab using a sterile spatula. BC was directly dispensed into the molds using its prefilled syringe. All procedures were performed under aseptic conditions in a laminar flow cabinet.

The molded specimens were incubated at 37 °C under 95% relative humidity for 48 hours to allow setting. After setting, the discs showed comparable consistency. The specimens were polished with fine polishing paper to obtain smooth and uniform surfaces and were measured with a vernier caliper. Each disc was rinsed with deionized water for 5 seconds, air dried, and sterilized under ultraviolet light for 30 minutes on each side. For mineralization assays, the discs were autoclaved at 121 °C for 20 minutes before use.

Leachates were prepared according to ISO 10993 12:2021 using a surface area to volume ratio of 3 cm^2^/mL. For pH and elemental release analyses, each group included three independently prepared specimens (n = 3). Each disc was immersed in deionized water for 48 hours in a sterile 5 mL plastic centrifuge tube and incubated at 37 °C. Measurements were performed at this single 48 hour time point. Release kinetics were not evaluated.

#### pH measurement and elemental release analysis.

The pH of each leachate prepared in deionized water was measured using a calibrated AB 15 Plus pH meter (Accumet Basic, Fisher Scientific, Pittsburgh, PA, USA). The pH meter was calibrated with standard buffer solutions at pH 4.00, 7.00, and 10.00 before measurement. Each sample was measured three times, and the average value was used for statistical analysis.

Elemental release from the tested materials was quantified using inductively coupled plasma optical emission spectroscopy (ICP OES; Optima 5300DV, PerkinElmer, San Francisco, CA, USA). Calcium, phosphorus, bismuth, lead, potassium, and magnesium were analyzed. Calibration was performed using commercially available multi element standard solutions covering the expected concentration ranges. Deionized water was used as the blank. Calibration curves were generated before sample analysis, and quality control was performed using calibration verification standards. Detection limits were instrument dependent and were within the range specified by the manufacturer.

The analyzed elements were selected based on their relevance to material composition and biological behavior. Calcium and phosphorus were included because they are associated with alkalinity, bioactivity, and mineralization. Bismuth was included because it is used as a radiopacifier in some tested materials. Lead was analyzed as a possible trace contaminant. Potassium and magnesium were measured to further characterize the ionic release profile of the materials.

#### Solubility.

The solubility of each material was evaluated according to ISO 6876:2001 for dental root canal sealing materials, as previously described [18]. Five independently prepared specimens were used for each group (n = 5). Each sample was weighed three times using an analytical balance, and the average value was recorded. These repeated measurements were considered technical replicates.

Stainless steel rings measuring 20 mm in inner diameter and 1.5 mm in height were used as molds. Each ring was filled with the test material, covered with a cellophane sheet, and compressed with a glass plate to obtain a flat surface. The specimens were incubated at 37 °C and 95% relative humidity for a period 50% longer than the setting time specified by the manufacturer: PM for 3 hours, MP for 3 hours, BR for 5.4 hours, and BC for 4 hours.

After setting, the specimens were removed from the molds and weighed three times using an analytical balance with an accuracy of 0.0001 g. Each specimen was placed in a pre weighed Petri dish containing 50 mL of deionized water and incubated at 37 °C and 95% relative humidity for 24 hours. After incubation, the specimens were gently rinsed with 2–3 mL of deionized water, and the rinsing solution was returned to the corresponding Petri dish. The dishes were dried in an oven at 110 °C, cooled to room temperature in a desiccator containing desiccant, and reweighed. Solubility was calculated from the difference between the final and initial mass of each Petri dish.

Because this test was conducted in deionized water, the results were interpreted as solubility under the present aqueous in vitro condition. They were not used alone to infer clinical performance.

### Biological properties

#### Cell culture.

Primary human periodontal ligament cells were isolated from periodontal tissues of two extracted maxillary first premolars. During isolation, tissues from both teeth were pooled, and donor specific cell lines were not established. Briefly, tissue explants were cultured in Dulbecco’s Modified Eagle Medium (DMEM; Gibco BRL) supplemented with 10% fetal bovine serum (FBS; Gibco BRL Life Technologies, Paisley, UK), 100 U/mL penicillin, and 100 U/mL streptomycin. Cells were maintained at 37 °C in a humidified atmosphere with 5% CO_2_. When cultures reached approximately 80% confluence, cells were detached using trypsin and subcultured. Cells from passages 3–8 were used. Because cells from the two teeth were pooled, donor specific variability was not assessed in this study.

#### Preparation of discs and leachates for biological assays.

Silicone molds measuring 9.5 mm in diameter and 1.5 mm in height were used to prepare discs of the tested materials. The molded specimens were incubated at 37 °C under 95% relative humidity for 48 hours to allow setting. The discs were then trimmed and processed as described above to obtain uniform dimensions. Each disc was rinsed with deionized water for 5 seconds and air dried.

Leachates were prepared according to ISO 10993 12:2021 using a surface area to volume ratio of 3 cm^2^/mL. Each disc was immersed in 5 mL of high glucose DMEM (Gibco BRL, Grand Island, NY, USA) in a sterile 5 mL centrifuge tube and incubated at 37 °C for either 24 hours or 48 hours. The resulting leachates were filtered through a 0.22 μm syringe filter for sterilization and stored at minus 20 °C until use. Each group included three independently prepared specimens.

#### Cytotoxicity.

Cell viability was assessed using the Cell Counting Kit 8 assay (CCK 8; Enzo Life Sciences Inc., Burlington, Ontario, Canada) according to the manufacturer’s protocol. hPDLCs were seeded in 96 well plates at a density of 6,000 cells per well and incubated for 24 hours. The culture medium was then replaced with 100 μL of serum free leachates from the tested materials diluted at 1:2, 1:4, or 1:10. Wells treated with serum free DMEM served as negative controls.

After 24, 48, or 72 hours of exposure, the leachates were removed. Then, 10 μL of CCK 8 solution mixed with 90 μL of serum free DMEM was added to each well. Plates were incubated for 2 hours at 37 °C, and absorbance was measured at 450 nm using an Elx808 Absorbance Reader (BioTek Instruments, Inc., Winooski, VT, USA). Cell viability ratios were calculated as previously described [18].

Cytotoxicity assays were performed in three independent experiments (n = 3). Within each experiment, technical replicate wells were averaged and treated as one independent data point. These assays were performed under serum free conditions, which were used to increase assay sensitivity but may not fully represent the physiological environment.

#### Mineralization.

hPDLCs were seeded into the lower chambers of 24 well Transwell plates with 0.4 μm pore inserts (Corning Inc., Corning, NY, USA) at a density of 2 × 10^5^ cells per well in high glucose DMEM supplemented with 10% FBS. Cells were incubated for 48 hours until confluence.

Sterilized material discs were placed into the Transwell inserts to allow indirect contact with the cells. The culture medium was replaced with osteogenic differentiation medium consisting of high glucose DMEM supplemented with 10% FBS, 100 U/mL penicillin, 100 U/mL streptomycin, 50 μg/mL ascorbic acid, 10 mmol/L β glycerophosphate, and 10 nmol/L dexamethasone (Sigma Aldrich, St. Louis, MO, USA). The medium was refreshed every 3 days. After 28 days of induction, the Transwell inserts were removed, and the cells in the lower chambers were fixed with 4% paraformaldehyde for Von Kossa staining to detect mineralized nodule formation. Control groups without sealer discs were included for baseline comparison.

Mineralization assays were conducted in three independent experiments (n = 3).

#### Inflammatory and matrix remodeling responses.

**Reverse transcription quantitative polymerase chain reaction:** Based on the cytotoxicity results, the 24 hour leachate at a 1:2 dilution was selected for subsequent gene expression experiments. hPDLCs were treated with diluted material leachates for 24 hours in serum free medium before RNA extraction.

Total RNA was extracted using the NucleoSpin RNA II kit (MACHEREY NAGEL GmbH & Co. KG, Düren, Germany), and RNA purity was assessed using the RNA/DNA Calculator (GeneQuant Pro; Amersham Biosciences, Little Chalfont, Buckinghamshire, UK). For cDNA synthesis, 1 μg of total RNA was reverse transcribed using reagents from Applied Biosystems (Life Technologies, Grand Island, NY, USA).

Quantitative PCR was performed in a total volume of 20 μL containing 10 μL SYBR Green, 1 μL of each primer at 5 μM, 4 μL distilled water, and 5 μL cDNA at 5 ng/μL. Reactions were run on a reverse transcription PCR instrument (Eppendorf AG, Hamburg, Germany) using the following thermal profile: initial denaturation at 94 °C for 3 minutes, followed by 40 cycles of 95 °C for 15 seconds and 60 °C for 20 seconds. Melt curve analysis was performed from 65 °C to 95 °C in 0.5 °C increments for 5 seconds each. GAPDH and ALG9 were used as reference genes. Non reverse transcribed RNA samples served as negative controls. Primer sequences and amplicon sizes are listed in Table 2.

**Table 2:** Primer sequences used for qRT PCR.

Gene	Forward primer (5’–3’)	Reverse primer (5’–3’)
*COL1A1*	AACCAAGGCTGCAACCTGGA	GGCTGAGTAGGGTACACGCAGG
*IL6*	CAACCTGAACCTTCCAAAGATG	TCTGGCTTGTTCCTCACTAC
*TGFβ1*	CAACGAAATCTATGACAAGTTCAAGCAG	CTTCTCGGAGCTCTGATGTG
*MMP1*	GCTAACAAATACTGGAGGTATGATG	GTCATGTGCTATCATTTTGGGA
*MMP2*	CTTCCAAGTCTGGAGCGATGT	TACCGTCAAAGGGGTATCCAT
*MMP3*	ATGATGAACAATGGACAAAGGA	GAGTGAAAGAGACCCAGGGA
*TIMP1*	CTGTGTCCCACCCCACC	GAACTTGGCCCTGATGACGA
*TIMP2*	ACATTTATGGCAACCCTATCAA	TCAGGCCCTTTGAACATCTTTA
*TIMP3*	AGGACGCCTTCTGCAAC	CTCCTTTACCAGCTTCTTCC
*TIMP4*	ACCTGTCCTTGGTGCAGA	TGTAGCAGGTGGTGATTTGG
*GAPDH*	CTTTGTCAAGCTCATTTCCTGGTA	GGCCATGAGGTCCACCA
*ALG9*	GAATGACCAGAATCTAGAAGAGCCA	TCTCATGGTGTCCAAATCCACTAAA

Gene expression analysis was performed in three independent experiments (n = 3). Technical replicates were averaged before statistical analysis.

**Western blot and ELISA:** For protein analysis, hPDLCs were treated with a 1:2 dilution of serum free material leachates for 48 hours. Conditioned media were collected and divided. Half of the medium, 500 μL per group, was stored at minus 80 °C for IL 6 quantification by ELISA. The remaining medium was centrifuged at 3,000 × g for 15 minutes at 4 °C to remove dead cells and debris. Proteins in the supernatant were precipitated with cold acetone on ice for 15 minutes and centrifuged again under the same conditions. The supernatant was discarded, and the protein pellets were resuspended in 1 × SDS sample buffer. Cell lysates were collected separately using 1 × SDS sample buffer.

Protein concentrations were determined using the Bio Rad DC protein assay (Bio Rad, Hemel Hempstead, UK). Equal amounts of protein were loaded onto SDS PAGE gels with molecular weight markers. After electrophoresis, proteins were transferred onto Hybond ECL nitrocellulose membranes (Amersham Biosciences) overnight at 4 °C. Membranes were blocked with Odyssey Blocking Buffer (LI COR Biosciences, Lincoln, NE, USA) and incubated overnight at 4 °C with primary antibodies. After washing with TBST, membranes were incubated with IRDye conjugated secondary antibodies at 1:20,000 for 1 hour at room temperature in the dark. Blots were visualized using the LI COR Odyssey Infrared Imaging System and quantified with Odyssey software version 3.0. GAPDH was used as a loading control.

IL 6 levels in culture supernatants were quantified using a human IL 6 ELISA kit (Thermo Fisher Scientific, Inc., Waltham, MA, USA). ELISA and Western blot analyses were performed in three independent experiments (n = 3). Technical replicates were averaged for ELISA. Western blot data were analyzed using normalized band intensities from independent experiments.

**Inhibition of cellular signaling pathways:** Previous studies have shown that MMP 1 expression in fibroblasts is regulated by p38, JNK, and ERK1/2 signaling pathways, which are activated through phosphorylation [19,20]. Because Western blot analysis showed increased MMP 1 secretion in the BR group, subsequent pathway experiments focused on BR. These experiments were performed to examine material specific signaling responses and were not intended to generalize mechanistic findings to the other tested sealers.

hPDLCs were treated with BR leachate at different time points and concentrations, and phosphorylation levels of ERK1/2, p38, and JNK were analyzed by Western blot.

To evaluate the involvement of these pathways, hPDLCs were pretreated with specific MAPK inhibitors for 1 hour before exposure to BR leachate. The inhibitors targeted ERK1/2, p38, and JNK. Cells were harvested after 48 hours for Western blot analysis to assess MMP 1 expression and pathway activation.

PM was used as the reference material because this study focused on calcium silicate based sealers and related biological responses. PM represents a prototype calcium silicate based material and provides a mechanistic reference for interpreting the behavior of newer calcium silicate based sealers. Although AH Plus is widely used as a clinical reference among epoxy resin based sealers, its distinct chemical composition and polymeric setting reaction make it less suitable for pathway experiments focused on calcium releasing materials.

### Statistical analysis

Statistical analyses were performed using SPSS version 16.0 (SPSS Inc., Chicago, IL, USA). Data are presented as mean ± standard deviation. For all analyses, n represents independent specimens or independent experimental runs. Technical replicates within each independent experiment were averaged and treated as one data point.

Before parametric testing, normality was assessed using the Shapiro Wilk test, and homogeneity of variance was assessed using Levene’s test. Parametric tests were applied only when these assumptions were met. For comparisons involving more than two groups, one way analysis of variance was used, followed by Tukey’s post hoc test for pairwise comparisons. Tukey’s test was used to control the family wise error rate for multiple comparisons. For comparisons between two groups, Student’s t test was used when appropriate.

If data had failed to meet assumptions of normality or homogeneity of variance, nonparametric tests such as the Kruskal Wallis test would have been considered. In this study, the datasets met the criteria for parametric analysis. The same statistical approach was applied to physicochemical and biological data, including pH, solubility, elemental release, cytotoxicity, gene expression, ELISA, and Western blot densitometry. A p value below 0.05 was considered statistically significant.

## Results

### pH

All material leachates showed highly alkaline pH values above 11 (Table 3). The pH values of the PM, MP, BC, and BR groups were significantly higher than that of the control medium (*p* < 0.05). No significant differences were detected among the four material groups.

**Table 3:** pH values of material leachates (48 h, deionized water).

	Control	PM	MP	BC	BR
**pH**	7.03 ± 0.05^a^	11.80 ± 0.20^b^	12.13 ± 0.15^b^	11.75 ± 0.22^b^	12.15 ± 0.21^b^

**Notes:**

• Data are presented as mean ± SD (n = 3 independent specimens).

• Different superscript letters indicate significant differences between groups (one way ANOVA with Tukey test, *p* < 0.05).

### Solubility

The solubility values differed significantly among the tested materials after immersion in deionized water (*p* < 0.05; Table 4). BC showed the highest solubility (19.63 ± 1.02%), followed by BR (14.67 ± 0.65%), MP (8.66 ± 1.35%), and PM (2.80 ± 0.70%). Under the present aqueous testing condition, only PM showed a solubility value below the 3% threshold specified in ISO 6876:2001. The solubility values of MP, BC, and BR exceeded this threshold. These findings should be interpreted in the context of the simplified deionized-water model used in this experiment and should not be taken as direct evidence of clinical performance.

**Table 4:** Solubility of tested materials (% mass loss).

	PM	MP	BC	BR
**Solubility (%)**	2.80 ± 0.70^a^	8.66 ± 1.35^b^	19.63 ± 1.02^d^	14.67 ± 0.65^c^

**Notes:**

• Data are presented as mean ± SD (n = 5 independent specimens).

• Different superscript letters indicate significant differences (one way ANOVA with Tukey test, *p* < 0.05).

• Measurements performed after 24 h immersion in deionized water according to ISO 6876:2001.

### Elemental release

Elemental release from the tested materials is summarized in Table 5. PM released the highest level of calcium among all groups (*p* < 0.05). MP released significantly more calcium than BC and BR (*p* < 0.05), whereas no significant difference was found between BC and BR.

**Table 5:** Elemental release from material leachates (48 h, mg/mL).

Element	PM	MP	BC	BR
Ca	13.57 ± 0.20^a^	9.71 ± 0.30^c^	6.76 ± 0.44^b^	6.65 ± 0.20^b^
P	21.72 ± 1.86^ab^	27.69 ± 1.39^a^	17.08 ± 2.99^b^	18.00 ± 1.83^b^
Bi	0.086 ± 0.004^a^	0.249 ± 0.024^b^	0.006 ± 0.001^c^	0.065 ± 0.014^ac^
Pb	0.16 ± 0.03^a^	0.076 ± 0.006^a^	0.125 ± 0.023^a^	0.187 ± 0.030^a^
K	82.18 ± 2.77^a^	84.21 ± 5.88^a^	84.19 ± 3.82^a^	64.67 ± 6.10^a^
Mg	20.92 ± 1.60^a^	13.13 ± 0.61^b^	12.25 ± 0.57^b^	12.84 ± 1.85^b^

**Notes:**

• Data are presented as mean ± SD (n = 3 independent specimens).

• Values represent concentrations in leachates after 48 h immersion.

• Different superscript letters indicate significant differences within each row (one way ANOVA with Tukey test, *p* < 0.05).

For phosphorus, MP showed significantly higher release than BC and BR (*p* < 0.05). No significant difference was observed between MP and PM, or among PM, BC, and BR. MP also released the highest amount of bismuth (*p* < 0.05). No significant differences were detected in lead or potassium release among the tested materials. Magnesium release was significantly higher in the PM group than in the MP, BC, and BR groups (*p* < 0.05), with no significant differences among the latter three groups.

### Cytoxicity

**Fig 1:**
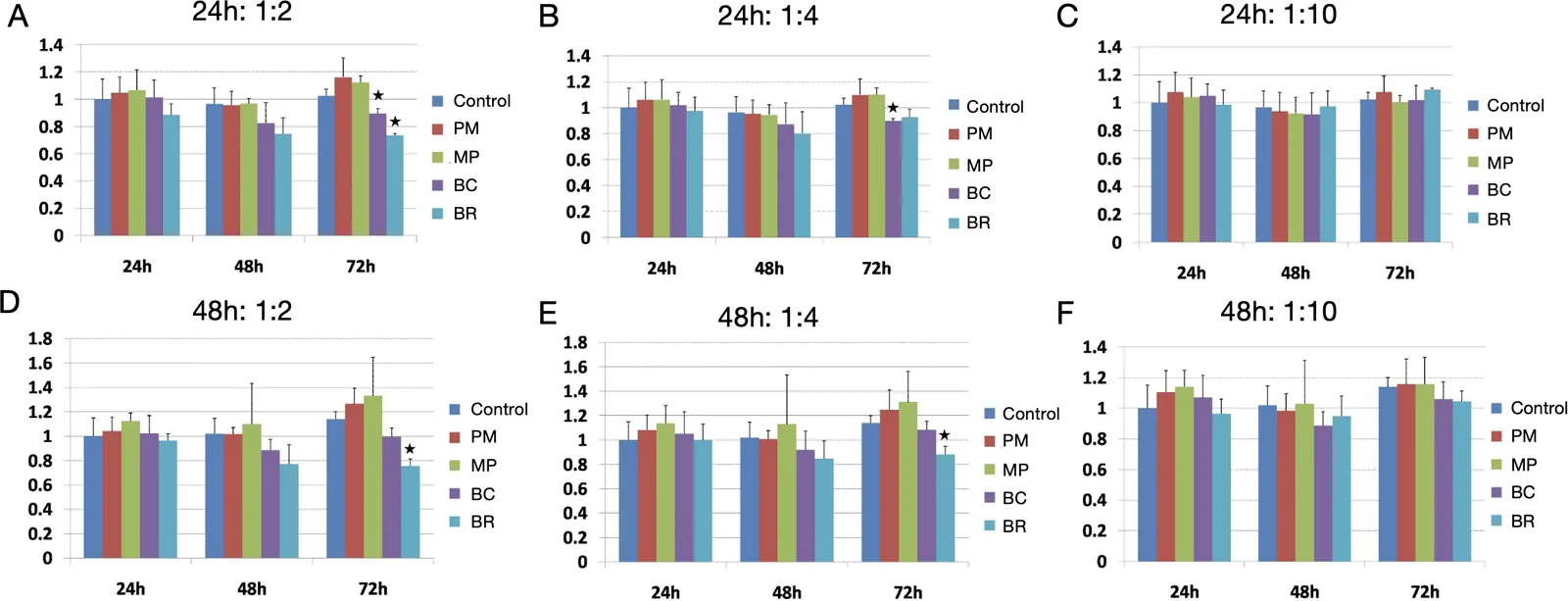
Cytotoxicity of material leachates in hPDLCs. Human periodontal ligament cells were exposed to 24 hour or 48 hour leachates from ProRoot MTA (PM), Grey MTA Plus (MP), Bio C Sealer (BC), and BioRoot RCS (BR) at 1:2, 1:4, and 1:10 dilutions. Cell viability was assessed using the CCK 8 assay after 24, 48, and 72 hours of exposure. Serum free DMEM was used as the control. Data are presented as mean ± SD from three independent experiments. Asterisks indicate significant differences compared with the control group at the same time point and dilution (*p* < 0.05).

The effects of 24-hour and 48-hour material leachates on hPDLC viability are shown in Fig 1. For 24-hour leachates, BC and BR at the 1:2 dilution significantly reduced cell viability after 72 hours of culture compared with the control (*p* < 0.05). BC at the 1:4 dilution also reduced cell viability at this time point (*p* < 0.05). For 48-hour leachates, BR significantly reduced cell viability at both 1:2 and 1:4 dilutions after 72 hours of culture (*p* < 0.05).

No marked reduction in cell viability was observed in the PM or MP groups under the tested conditions. The cytotoxic effects observed for BC and BR were more evident at higher leachate concentrations and longer exposure times.

### Mineralization

**Fig 2:**
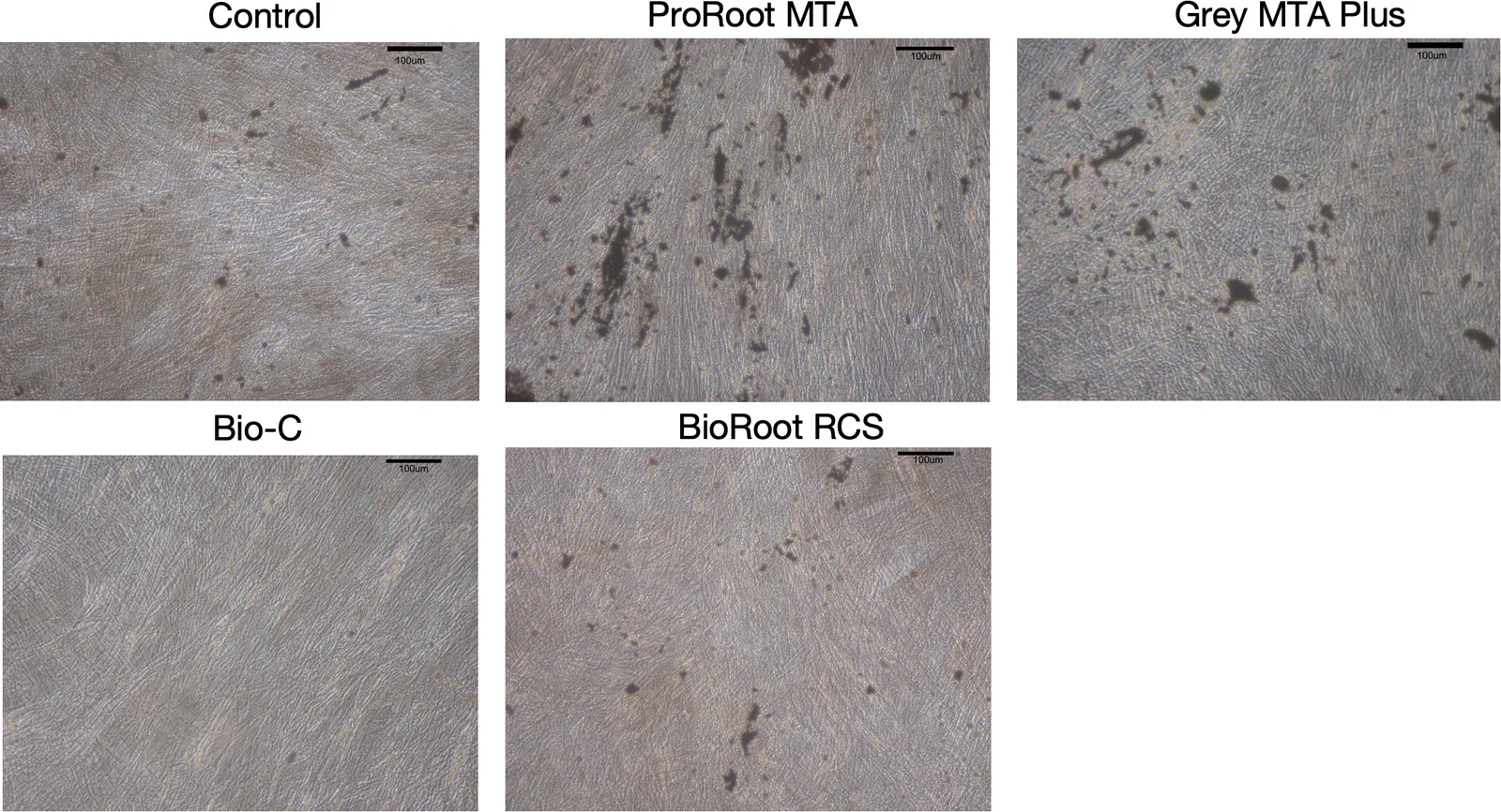
Mineralized nodule formation in hPDLCs after exposure to material discs. Human periodontal ligament cells were cultured in osteogenic differentiation medium for 28 days with material discs placed in Transwell inserts. Mineralized nodules were detected by Von Kossa staining. The control group was cultured without material discs. PM and MP showed greater mineral deposition than the control, whereas BR and BC showed less evident mineralized nodule formation under the tested conditions.

Mineralized nodule formation after 28 days of osteogenic induction is shown in Fig 2. Compared with the control group, more mineral deposition was observed in the PM and MP groups. The BR group showed fewer mineralized nodules than PM and MP, while only limited mineral deposition was observed in the BC group.

### Effects of material leachates on gene expression in hPDLCs

**Fig 3:**
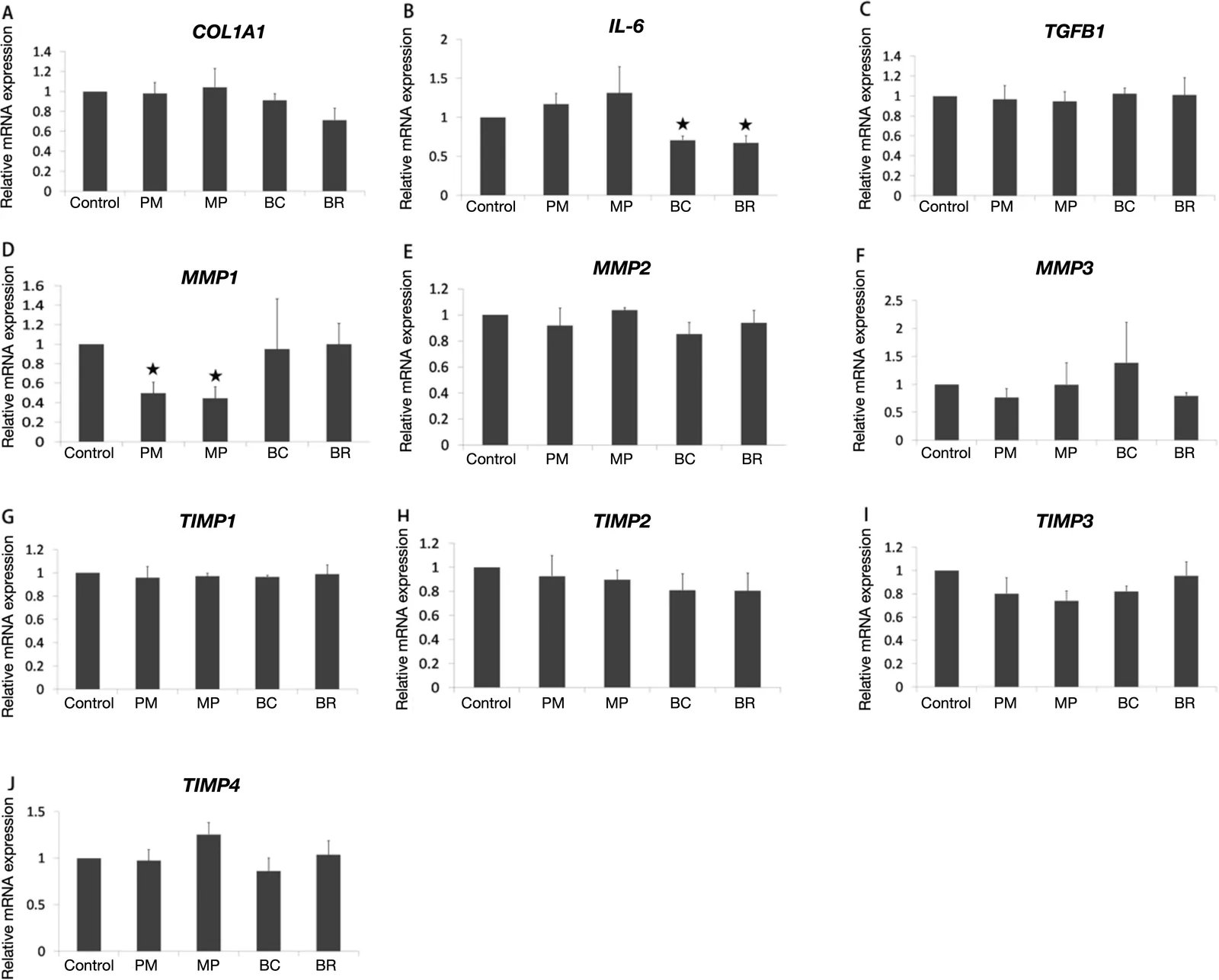
Gene expression in hPDLCs after exposure to material leachates. Relative mRNA expression levels of *COL1A1*, *IL6*, *TGFB1*, *MMP1*, *MMP2*, *MMP3*, *TIMP1*, *TIMP2*, *TIMP3*, and *TIMP4* were measured by qRT PCR after exposure to leachates from PM, MP, BC, and BR. *GAPDH* and *ALG9* were used as reference genes. Data are presented as mean ± SD from three independent experiments. Asterisks indicate significant differences compared with the control group (*p* < 0.05).

The mRNA expression levels of *COL1A1*, *TGFB1*, *IL6*, *MMP1*, *MMP2*, *MMP3*, *TIMP1*, *TIMP2*, *TIMP3*, and *TIMP4* were assessed after exposure to material leachates (Fig 3). IL6 expression was significantly reduced in the BC and BR groups compared with the control (*p* < 0.05). MMP1 expression was significantly downregulated in the PM and MP groups (*p* < 0.05). No significant differences were detected for *COL1A1*, *TGFB1*, *MMP2*, *MMP3*, *TIMP1*, *TIMP2*, *TIMP3*, or *TIMP4* among the tested groups.

### Verification of MMP-1 and IL-6 protein levels

**Fig 4:**
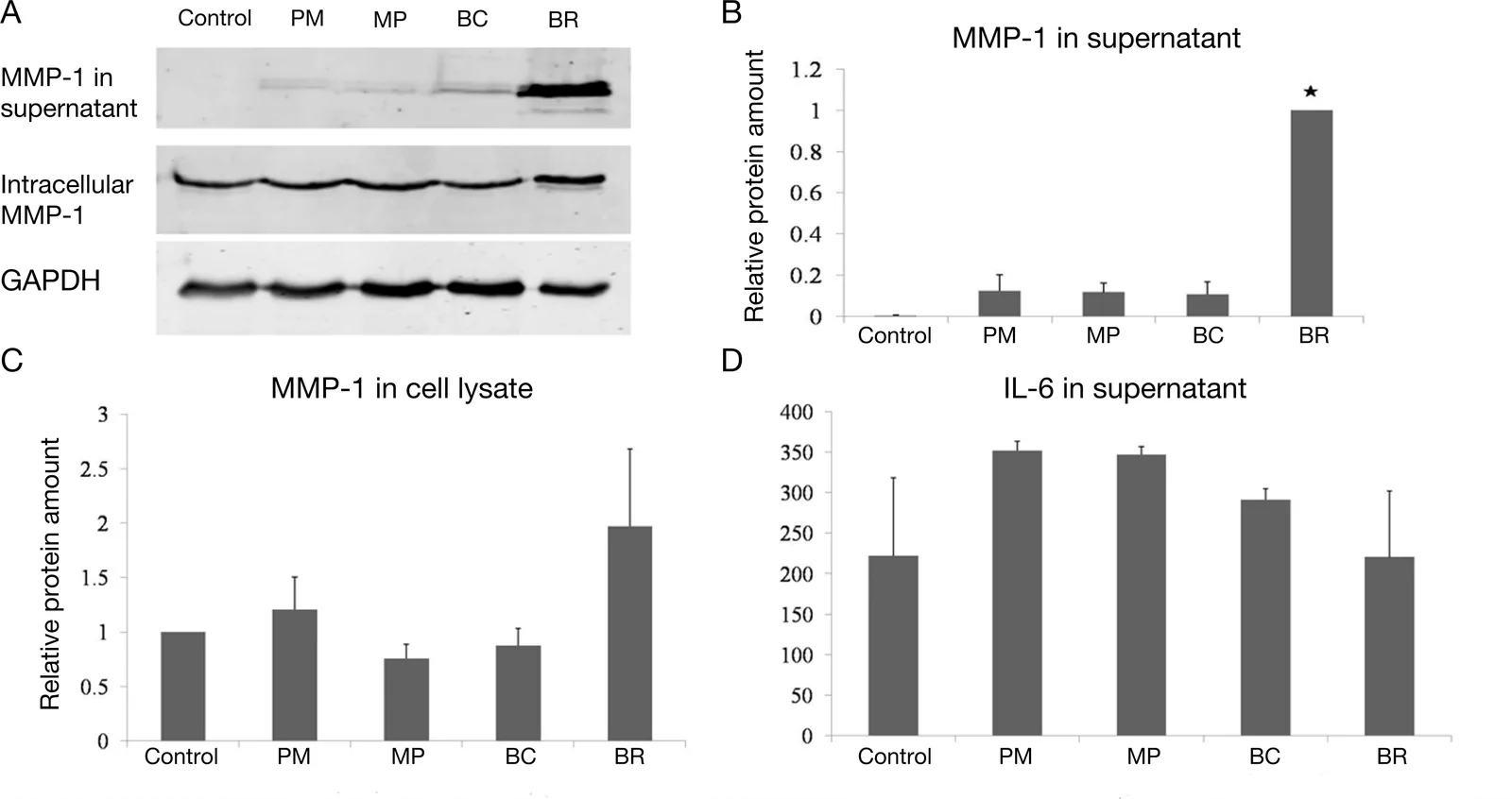
MMP 1 and IL 6 protein levels in hPDLC cultures after exposure to material leachates. MMP 1 protein levels in culture supernatants and cell lysates were assessed by Western blot after exposure to leachates from PM, MP, BC, and BR. IL 6 levels in culture supernatants were measured by ELISA. GAPDH was used as the loading control for cell lysates. Data are presented as mean ± SD from three independent experiments. Asterisks indicate significant differences compared with the control group (*p* < 0.05).

MMP-1 protein levels were assessed by Western blot, and IL-6 protein levels were measured by ELISA (Fig 4). In the culture supernatant, MMP-1 protein levels were significantly increased in the BR group compared with the control (*p* < 0.05). No significant increase in secreted MMP-1 was detected in the PM, MP, or BC groups. In cell lysates, MMP-1 protein levels did not differ significantly among groups.

Although IL6 mRNA expression was reduced in the BC and BR groups, IL-6 protein levels in the culture supernatant were not significantly different among the groups. These results indicate that the transcriptional change in IL6 was not accompanied by a corresponding change in IL-6 protein secretion under the present experimental conditions. Because BR showed a distinct increase in secreted MMP-1, subsequent pathway experiments were focused on this material.

### Modulation of MAPK signaling pathways by BR leachate

**Fig 5:**
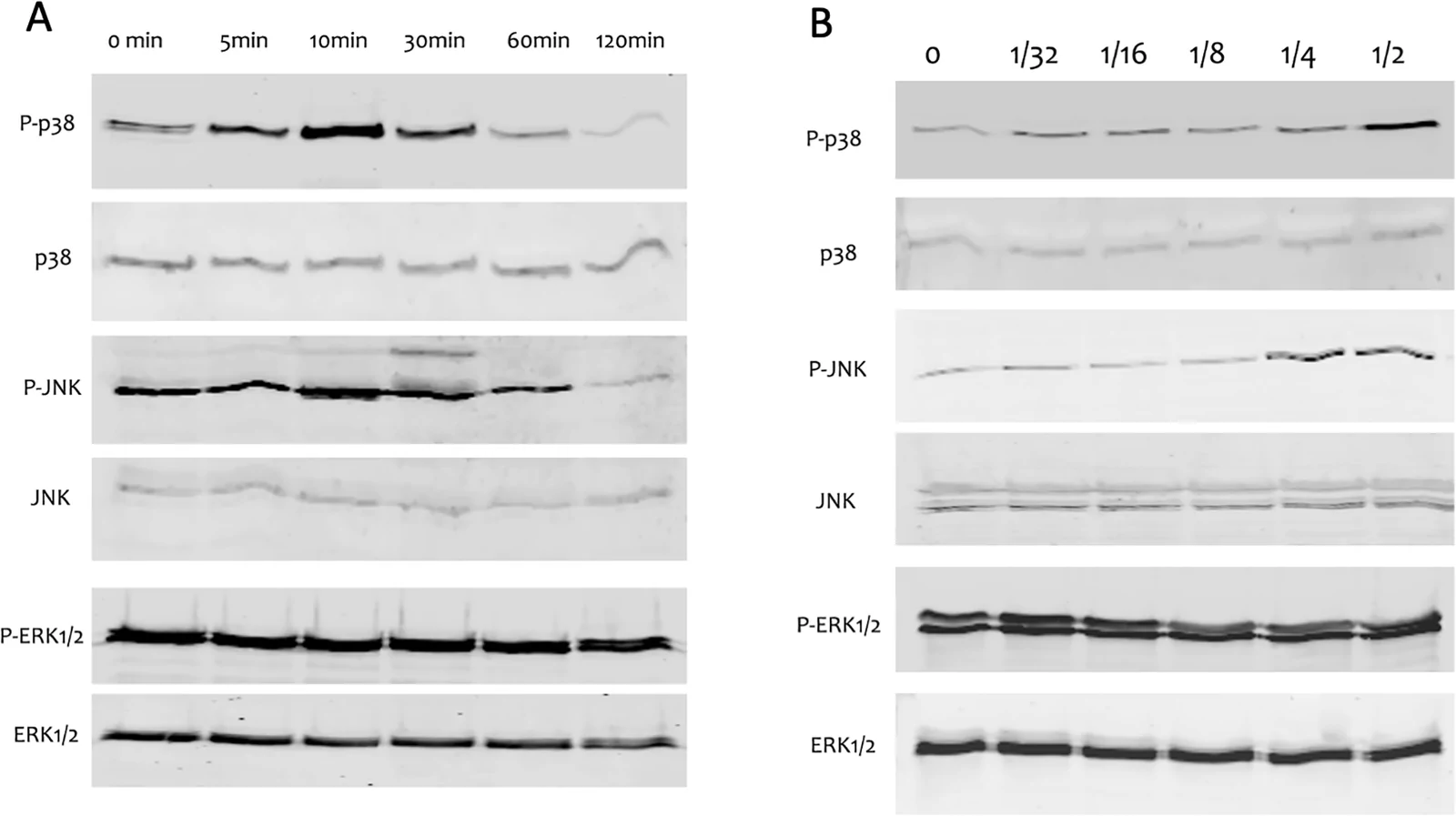
MAPK pathway activation in hPDLCs treated with BioRoot RCS leachate. Western blot analysis was used to assess phosphorylation of p38, JNK, and ERK1/2 in hPDLCs treated with BR leachate. GAPDH was used as the loading control. (A) Time course analysis after treatment with BR leachate at a 1:2 dilution. (B) Concentration response analysis after 10 minutes of treatment with different BR leachate dilutions. These experiments were performed only with BR and should be interpreted as material specific.

MAPK pathway activation was examined in hPDLCs treated with BR leachate. Western blot analysis showed increased phosphorylation of p38, JNK, and ERK1/2 after exposure to BR leachate (Fig 5). Phosphorylation of p38 and JNK was most evident at 10 minutes. The response also varied with leachate concentration. Because these pathway experiments were performed only with BR, the findings should be interpreted as material-specific and should not be generalized to PM, MP, or BC.

### BR leachate-induced MMP-1 secretion and MAPK inhibition

**Fig 6:**
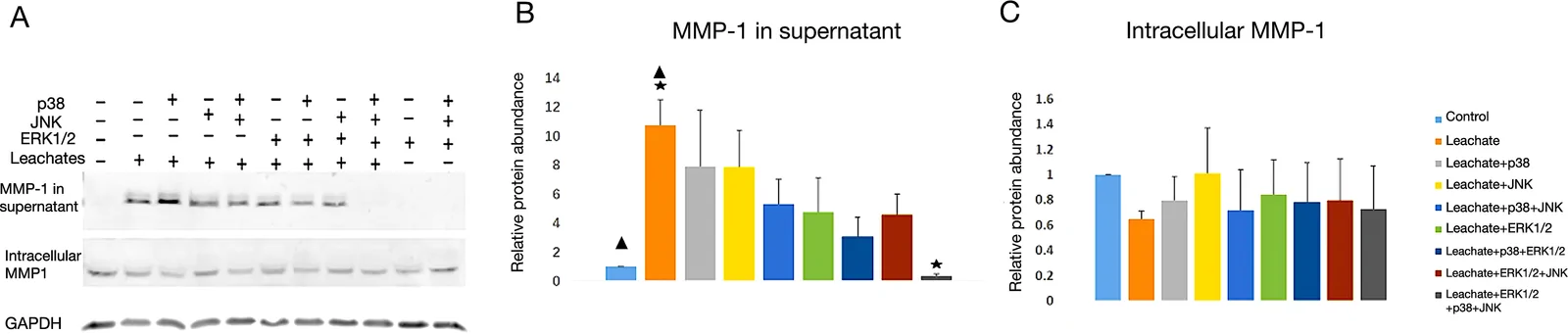
Effects of MAPK pathway inhibition on BR associated MMP 1 secretion. hPDLCs were pretreated with inhibitors targeting ERK1/2 (PD184352), p38 (SB203580), and JNK (SP600125) before exposure to BR leachate. MMP 1 protein levels in culture supernatants and cell lysates were assessed by Western blot. GAPDH was used as the loading control for cell lysates. Data are presented as mean ± SD from three independent experiments. The same symbol indicates a statistically significant difference between the indicated groups (*p* < 0.05).

To examine whether MAPK signaling was involved in BR-associated MMP-1 secretion, hPDLCs were pretreated with inhibitors of ERK1/2, p38, and JNK before exposure to BR leachate. Combined inhibition of these pathways significantly reduced MMP-1 protein levels in the culture supernatant (*p* < 0.05; Fig 6A, B). No significant differences were observed in MMP-1 levels in cell lysates (Fig 6A, C). These results suggest that ERK1/2, p38, and JNK signaling contribute to BR leachate-associated MMP-1 secretion in hPDLCs. The data support a material-specific effect on pathways related to matrix remodeling, rather than evidence of a generalized inflammatory response.

## Discussion

With the increasing clinical use of calcium silicate based root canal sealers, it is important to evaluate both their physicochemical behavior and their biological responses under controlled conditions. In the present study, the solubility, pH, ion release, cytocompatibility, mineralization, and selected inflammatory and matrix remodeling responses of three calcium silicate based sealers, MP, BR, and BC, were examined using hPDLCs, with PM included as a mechanistic reference material.

PM is not used clinically as a conventional root canal sealer. Its inclusion in this study was intended to provide a reference for interpreting material behavior within the same calcium silicate system. As one of the earliest calcium silicate based materials, PM has been extensively characterized and has shown favorable biocompatibility and bioactivity in various applications [6–8]. It is therefore commonly used as a reference material in studies of calcium silicate based systems [21]. Comparisons with PM in the present study should be interpreted in this context rather than as a direct clinical comparison.

Solubility is a key property for root canal sealers, as excessive material loss may affect sealing ability and long term stability [22]. According to ISO 6876:2001, solubility should not exceed 3% after 24 hours of water immersion. In the present study, MP, BR, and BC exceeded this threshold, whereas PM remained below it. Similar findings have been reported for calcium silicate based sealers [23]. However, it should be noted that the conventional solubility test is performed in deionized water and does not reproduce the ionic composition of clinical fluids. Previous studies have shown that immersion in phosphate containing solutions such as PBS may alter solubility and precipitation behavior [14,24], although results are not entirely consistent across studies [14,25]. Differences in setting conditions may also contribute to variability, and incomplete setting has been reported to influence solubility outcomes [26]. Therefore, the present results should be interpreted as solubility under a simplified aqueous condition rather than as a direct indicator of clinical performance [27].

The higher solubility observed for BR and BC may be related to their formulation and may contribute to increased release of material components. In calcium silicate based materials, dissolution is closely associated with ion release and local changes in pH [22,28–30]. These processes may influence cellular responses. In the present study, BC and BR showed reduced cell viability at higher concentrations and longer exposure times. These effects may be related, at least in part, to the amount of released components. However, the current data do not allow separation of dissolution driven effects from composition specific effects, and this relationship should be interpreted with caution.

PM and MP released higher levels of calcium and were associated with more mineralized nodule formation. These findings are consistent with previous reports that calcium silicate based materials with higher calcium release may promote mineral deposition [28–30]. Calcium ion release and alkaline conditions are known to support apatite formation and mineralization [15,29]. The higher calcium release observed in PM and MP is consistent with their tricalcium silicate rich composition. In addition, both materials contain bismuth oxide as a radiopacifier, and MP showed higher bismuth release. Previous work has suggested that bismuth containing materials may influence calcium phosphate deposition [31]. The mineralization observed in the present study is therefore likely related to the combined effects of ion release and alkalinity rather than a single component.

All tested materials produced alkaline leachates, with pH values above 11. Such alkaline conditions have been reported for calcium silicate based sealers [28–30,32] and are associated with antimicrobial activity, apatite formation, and mineralized tissue deposition [29,33]. Alkalinity may also facilitate the release of bioactive molecules from dentin [34]. At the same time, high pH may affect cell viability depending on exposure conditions. Therefore, alkalinity should be considered in relation to both potential bioactivity and cytotoxicity.

hPDLCs were selected because they are among the cell populations that may be exposed to sealer components in the periapical region and are involved in tissue repair and remodeling [35]. In the present study, PM and MP did not markedly reduce cell viability under the tested conditions, whereas BC and BR reduced cell viability after prolonged exposure. These findings are generally consistent with previous reports showing that calcium silicate based materials exhibit variable cytocompatibility depending on formulation and experimental conditions [36–38]. Differences between studies may be related to cell type, exposure conditions, and assay methods.

It should be noted that cytotoxicity assays in the present study were performed under serum free conditions. This approach is commonly used to increase assay sensitivity and reduce variability, but it does not replicate the in vivo environment. Serum proteins may bind or dilute released components, which could reduce cytotoxic effects. Therefore, the observed responses may represent a more stringent condition than would occur clinically. In addition, hPDLCs were pooled from two donors, and donor specific variability was not assessed. Periodontal ligament cells from different individuals may differ in biological responses, and this limitation should be considered when interpreting the results.

With respect to inflammatory and matrix remodeling responses, no significant changes were observed in the expression of *COL1A1*, *TGFB1*, *MMP2*, *MMP3*, or *TIMPs*. *IL6* mRNA expression was reduced in the BC and BR groups, but IL 6 protein levels in the culture supernatant were not significantly different. This discrepancy indicates that changes at the mRNA level were not reflected at the protein level under the present conditions. Such differences may be related to post transcriptional regulation, differences in protein secretion, or timing of measurement.

In contrast, BR increased MMP 1 protein secretion, although *MMP1* mRNA expression was not increased. MMP 1 is involved in extracellular matrix degradation and is associated with tissue remodeling processes [39,40]. The increase in MMP 1 secretion in the BR group suggests an effect on matrix remodeling related pathways rather than a generalized inflammatory response. Previous studies have reported that BR may modulate inflammatory responses, including cytokine production, depending on the experimental model [10,41]. The present findings should therefore be interpreted in the context of the specific markers and conditions used in this study.

The lack of correspondence between IL 6 protein levels and MMP 1 secretion suggests that inflammatory cytokine signaling and matrix remodeling may be regulated through different mechanisms. MMP 1 expression can be regulated by signaling pathways such as MAPK that are not directly dependent on IL 6 [19,20]. This may explain the observed pattern in the present study.

MAPK signaling pathways, including p38, JNK, and ERK1/2, have been implicated in the regulation of MMP 1 expression in fibroblastic cells [19,20]. In the present study, BR leachate increased phosphorylation of these pathways, and inhibition of these pathways reduced MMP 1 secretion. These findings support an association between BR exposure and activation of MAPK pathways involved in MMP 1 regulation. However, these experiments were performed only for BR, and the results should be interpreted as material specific.

The relatively high solubility of BR may contribute to its effects on MAPK activation and MMP 1 secretion. Increased release of material components may enhance cellular exposure to bioactive ions or other substances [42–44]. However, the specific components responsible for these effects were not identified in the present study, and further work is required to clarify the underlying mechanisms.

Several limitations should be considered. Solubility testing was performed in deionized water and does not reflect the complexity of clinical conditions. Biological assays were conducted in vitro and do not account for interactions with immune cells or tissue level responses. Cells were pooled from two donors, and donor variability was not assessed. Cytotoxicity assays were performed under serum free conditions. In addition, mechanistic pathway analysis was limited to BR and was not performed for the other materials.

## Conclusion

Within the limitations of this in vitro study, PM and MP showed higher calcium release and were associated with increased mineralized nodule formation, whereas BC and BR showed higher solubility. BC and BR reduced cell viability at higher concentrations and longer exposure times, and BR increased MMP-1 protein secretion. These findings may be related to differences in solubility and ion release, but the relative contributions of dissolution and material composition cannot be determined. MAPK activation observed in the BR group suggests involvement in MMP-1 regulation rather than a generalized inflammatory response. Given the in vitro design, including the use of deionized water, pooled cells, and serum-free conditions, the results should be interpreted with caution. Further studies under more clinically relevant conditions are required.

## Supporting information

S1 File Raw data. (XLSX)

S2 File Raw images. (PDF)

## References

[1] Li G-H, Niu L-N, Zhang W, Olsen M, De-Deus G, Eid AA, et al. Ability of new obturation materials to improve the seal of the root canal system: a review. Acta Biomater. 2014;10(3):1050–63. doi: 10.1016/j.actbio.2013.11.015 24321349 PMC3939610

[2] Liu H, Li H, Zhang L, Wang Z, Qian J, Yu M, et al. In vitro evaluation of the antibacterial effect of four root canal sealers on dental biofilms. Clin Oral Investig. 2022;26(6):4361–8. doi: 10.1007/s00784-022-04399-9 35137277

[3] Liu H, Lai WWM, Hieawy A, Gao Y, von Bergmann H, Haapasalo M, et al. Micro-computed tomographic evaluation of the quality of root canal fillings in mandibular molars after obturation for 54 months. J Endod. 2021;47(11):1783–9. doi: 10.1016/j.joen.2021.08.015 34492231

[4] Braga JM, Oliveira RR, de Castro Martins R, Vieira LQ, Sobrinho APR. Assessment of the cytotoxicity of a mineral trioxide aggregate-based sealer with respect to macrophage activity. Dent Traumatol. 2015;31(5):390–5. doi: 10.1111/edt.12190 26086068

[5] Tepel J, Darwisch el Sawaf M, Hoppe W. Reaction of inflamed periapical tissue to intracanal medicaments and root canal sealers. Endod Dent Traumatol. 1994;10(5):233–8. doi: 10.1111/j.1600-9657.1994.tb00076.x 7843066

[6] Torabinejad M, Parirokh M, Dummer PMH. Mineral trioxide aggregate and other bioactive endodontic cements: an updated overview - part II: other clinical applications and complications. Int Endod J. 2018;51(3):284–317. doi: 10.1111/iej.12843 28846134

[7] Parirokh M, Torabinejad M. Mineral trioxide aggregate: a comprehensive literature review--Part I: chemical, physical, and antibacterial properties. J Endod. 2010;36(1):16–27. doi: 10.1016/j.joen.2009.09.006 20003930

[8] de Oliveira NG, de Souza Araújo PR, da Silveira MT, Sobral APV, Carvalho M de V. Comparison of the biocompatibility of calcium silicate-based materials to mineral trioxide aggregate: Systematic review. Eur J Dent. 2018;12(2):317–26. doi: 10.4103/ejd.ejd_347_17 29988207 PMC6004803

[9] Siboni F, Taddei P, Prati C, Gandolfi MG. Properties of NeoMTA Plus and MTA Plus cements for endodontics. Int Endod J. 2017;50 Suppl 2:e83–94. doi: 10.1111/iej.12787 28452115

[10] Jeanneau C, Giraud T, Laurent P, About I. BioRoot RCS extracts modulate the early mechanisms of periodontal inflammation and regeneration. J Endod. 2019;45(8):1016–23. doi: 10.1016/j.joen.2019.04.003 31160081

[11] Gaudin A, Tolar M, Peters OA. Cytokine production and cytotoxicity of calcium silicate-based sealers in 2- and 3-dimensional cell culture models. J Endod. 2020;46(6):818–26. doi: 10.1016/j.joen.2020.03.011 32331837

[12] Zordan-Bronzel CL, Esteves Torres FF, Tanomaru-Filho M, Chávez-Andrade GM, Bosso-Martelo R, Guerreiro-Tanomaru JM. Evaluation of physicochemical properties of a new calcium silicate-based sealer, Bio-C Sealer. J Endod. 2019;45(10):1248–52. doi: 10.1016/j.joen.2019.07.006 31447172

[13] Alves Silva EC, Tanomaru-Filho M, da Silva GF, Delfino MM, Cerri PS, Guerreiro-Tanomaru JM. Biocompatibility and bioactive potential of new calcium silicate-based endodontic sealers: Bio-C Sealer and Sealer Plus BC. J Endod. 2020;46(10):1470–7. doi: 10.1016/j.joen.2020.07.011 32682789

[14] Torres FFE, Zordan-Bronzel CL, Guerreiro-Tanomaru JM, Chávez-Andrade GM, Pinto JC, Tanomaru-Filho M. Effect of immersion in distilled water or phosphate-buffered saline on the solubility, volumetric change and presence of voids within new calcium silicate-based root canal sealers. Int Endod J. 2020;53(3):385–91. doi: 10.1111/iej.13225 31566768

[15] Gandolfi MG, Taddei P, Modena E, Siboni F, Prati C. Biointeractivity-related versus chemi/physisorption-related apatite precursor-forming ability of current root end filling materials. J Biomed Mater Res B Appl Biomater. 2013;101(7):1107–23. doi: 10.1002/jbm.b.32920 23559495

[16] da Fonseca TS, da Silva GF, Tanomaru-Filho M, Sasso-Cerri E, Guerreiro-Tanomaru JM, Cerri PS. In vivo evaluation of the inflammatory response and IL-6 immunoexpression promoted by Biodentine and MTA Angelus. Int Endod J. 2016;49(2):145–53. doi: 10.1111/iej.12435 25644518

[17] Yang X, Tian J, Li M, Chen W, Liu H, Wang Z, et al. Biocompatibility of a new calcium silicate-based root canal sealer mediated via the modulation of macrophage polarization in a rat model. Materials (Basel). 2022;15(5):1962. doi: 10.3390/ma15051962 35269193 PMC8911908

[18] Chen B, Haapasalo M, Mobuchon C, Li X, Ma J, Shen Y. Cytotoxicity and the effect of temperature on physical properties and chemical composition of a new calcium silicate-based root canal sealer. J Endod. 2020;46(4):531–8. doi: 10.1016/j.joen.2019.12.009 32081458

[19] Brauchle M, Glück D, Di Padova F, Han J, Gram H. Independent role of p38 and ERK1/2 mitogen-activated kinases in the upregulation of matrix metalloproteinase-1. Exp Cell Res. 2000;258(1):135–44. doi: 10.1006/excr.2000.4913 10912795

[20] Ni S, Li C, Xu N, Liu X, Wang W, Chen W, et al. Follistatin-like protein 1 induction of matrix metalloproteinase 1, 3 and 13 gene expression in rheumatoid arthritis synoviocytes requires MAPK, JAK/STAT3 and NF-κB pathways. J Cell Physiol. 2018;234(1):454–63. doi: 10.1002/jcp.26580 29932210

[21] Singh G, Gupta I, Elshamy FMM, Boreak N, Homeida HE. In vitro comparison of antibacterial properties of bioceramic-based sealer, resin-based sealer and zinc oxide eugenol based sealer and two mineral trioxide aggregates. Eur J Dent. 2016;10(3):366–9. doi: 10.4103/1305-7456.184145 27403055 PMC4926590

[22] Gandolfi MG, Siboni F, Primus CM, Prati C. Ion release, porosity, solubility, and bioactivity of MTA Plus tricalcium silicate. J Endod. 2014;40(10):1632–7. doi: 10.1016/j.joen.2014.03.025 25260736

[23] Colombo M, Poggio C, Dagna A, Meravini M-V, Riva P, Trovati F, et al. Biological and physico-chemical properties of new root canal sealers. J Clin Exp Dent. 2018;10(2):e120–6. doi: 10.4317/jced.54548 29670728 PMC5899788

[24] Urban K, Neuhaus J, Donnermeyer D, Schäfer E, Dammaschke T. Solubility and pH Value of 3 different root canal sealers: A long-term investigation. J Endod. 2018;44(11):1736–40. doi: 10.1016/j.joen.2018.07.026 30243663

[25] Zhou H, Shen Y, Zheng W, Li L, Zheng Y, Haapasalo M. Physical properties of 5 root canal sealers. J Endod. 2013;39(10):1281–6. doi: 10.1016/j.joen.2013.06.012 24041392

[26] Lee JK, Kwak SW, Ha J-H, Lee W, Kim H-C. Physicochemical properties of epoxy resin-based and bioceramic-based root canal sealers. Bioinorg Chem Appl. 2017;2017:2582849. doi: 10.1155/2017/2582849 28210204 PMC5292198

[27] Siboni F, Taddei P, Zamparini F, Prati C, Gandolfi MG. Properties of BioRoot RCS, a tricalcium silicate endodontic sealer modified with povidone and polycarboxylate. Int Endod J. 2017;50 Suppl 2:e120–36. doi: 10.1111/iej.12856 28881478

[28] Souza LC, Neves GST, Kirkpatrick T, Letra A, Silva R. Physicochemical and Biological Properties of AH Plus Bioceramic. J Endod. 2023;49(1):69–76. doi: 10.1016/j.joen.2022.10.009 36279961

[29] Chen JH, Raman V, Kuehne SA, Camilleri J, Hirschfeld J. Chemical, antibacterial, and cytotoxic properties of four different endodontic sealer leachates over time. J Endod. 2024;50(11):1612–21. doi: 10.1016/j.joen.2024.08.015 39197739

[30] Mann A, Zeng Y, Kirkpatrick T, van der Hoeven R, Silva R, Letra A, et al. Evaluation of the physicochemical and biological properties of EndoSequence BC Sealer HiFlow. J Endod. 2022;48(1):123–31. doi: 10.1016/j.joen.2021.10.001 34678358

[31] Pazarçeviren AE, Evis Z, Keskin D, Tezcaner A. Resorbable PCEC/gelatin-bismuth doped bioglass-graphene oxide bilayer membranes for guided bone regeneration. Biomed Mater. 2019;14(3):035018. doi: 10.1088/1748-605X/ab007b 30665204

[32] Liu H, Chen C, Wang Z, Shen Y. Correlations among antibacterial efficacy in dentinal tubules, pH, and calcium ion release of 5 premixed calcium silicate-based sealers in a Novel ex vivo model. J Endod. 2025;51(11):1599–608. doi: 10.1016/j.joen.2025.05.026 40482706

[33] Holland R, Gomes JEF, Cintra LTA, Queiroz IODA, Estrela C. Factors affecting the periapical healing process of endodontically treated teeth. J Appl Oral Sci. 2017;25(5):465–76. doi: 10.1590/1678-7757-2016-0464 29069143 PMC5804382

[34] Graham L, Cooper PR, Cassidy N, Nor JE, Sloan AJ, Smith AJ. The effect of calcium hydroxide on solubilisation of bio-active dentine matrix components. Biomaterials. 2006;27(14):2865–73. doi: 10.1016/j.biomaterials.2005.12.020 16427123

[35] Tomokiyo A, Wada N, Maeda H. Periodontal ligament stem cells: regenerative potency in periodontium. Stem Cells Dev. 2019;28(15):974–85. doi: 10.1089/scd.2019.0031 31215350

[36] Eid AA, Gosier JL, Primus CM, Hammond BD, Susin LF, Pashley DH, et al. In vitro biocompatibility and oxidative stress profiles of different hydraulic calcium silicate cements. J Endod. 2014;40(2):255–60. doi: 10.1016/j.joen.2013.07.009 24461414 PMC3904134

[37] Kim M, Yang W, Kim H, Ko H. Comparison of the biological properties of ProRoot MTA, OrthoMTA, and Endocem MTA cements. J Endod. 2014;40(10):1649–53. doi: 10.1016/j.joen.2014.04.013 25052144

[38] López-García S, Lozano A, García-Bernal D, Forner L, Llena C, Guerrero-Gironés J, et al. Biological effects of new hydraulic materials on human periodontal ligament stem cells. J Clin Med. 2019;8(8):1216. doi: 10.3390/jcm8081216 31416236 PMC6722926

[39] Hosokawa I, Hosokawa Y, Shindo S, Ozaki K, Matsuo T. Melatonin inhibits CXCL10 and MMP-1 production in IL-1β-stimulated human periodontal ligament cells. Inflammation. 2016;39(4):1520–6. doi: 10.1007/s10753-016-0386-3 27271323

[40] Liu H, Yu J, Hieawy A, Hu Z, Tay FR, Shen Y. Design and evaluation of an MMP-9-responsive hydrogel for vital pulp therapy. J Dent. 2024;146:105020. doi: 10.1016/j.jdent.2024.105020 38670329

[41] Gaudin A, Tolar M, Peters OA. Cytokine production and cytotoxicity of calcium silicate-based sealers in 2- and 3-dimensional cell culture models. J Endod. 2020;46(6):818–26. doi: 10.1016/j.joen.2020.03.011 32331837

[42] Chang S-W, Lee S-Y, Ann H-J, Kum K-Y, Kim E-C. Effects of calcium silicate endodontic cements on biocompatibility and mineralization-inducing potentials in human dental pulp cells. J Endod. 2014;40(8):1194–200. doi: 10.1016/j.joen.2014.01.001 25069932

[43] Mora A, García-Bernal D, Rodríguez-Lozano FJ, Sanz JL, Forner L, Ghilotti J, et al. Biocompatibility, bioactivity and immunomodulatory properties of three calcium silicate-based sealers: an in vitro study on hPDLSCs. Clin Oral Investig. 2024;28(8):416. doi: 10.1007/s00784-024-05812-1 38969964 PMC11226489

[44] Rathinam E, Govindarajan S, Rajasekharan S, Declercq H, Elewaut D, De Coster P, et al. The calcium dynamics of human dental pulp stem cells stimulated with tricalcium silicate-based cements determine their differentiation and mineralization outcome. Sci Rep. 2021;11(1):645. doi: 10.1038/s41598-020-80096-5 33436827 PMC7804324

