# Supplementary material for: Physicochemical and biological properties of three calcium silicate-based root canal sealers
Source: PLoS One. 2026 Sep 24;21(9):e0359096. doi: 10.1371/journal.pone.0359096 (PMC13606944; doi:10.1371/journal.pone.0359096)

Fig. 4A

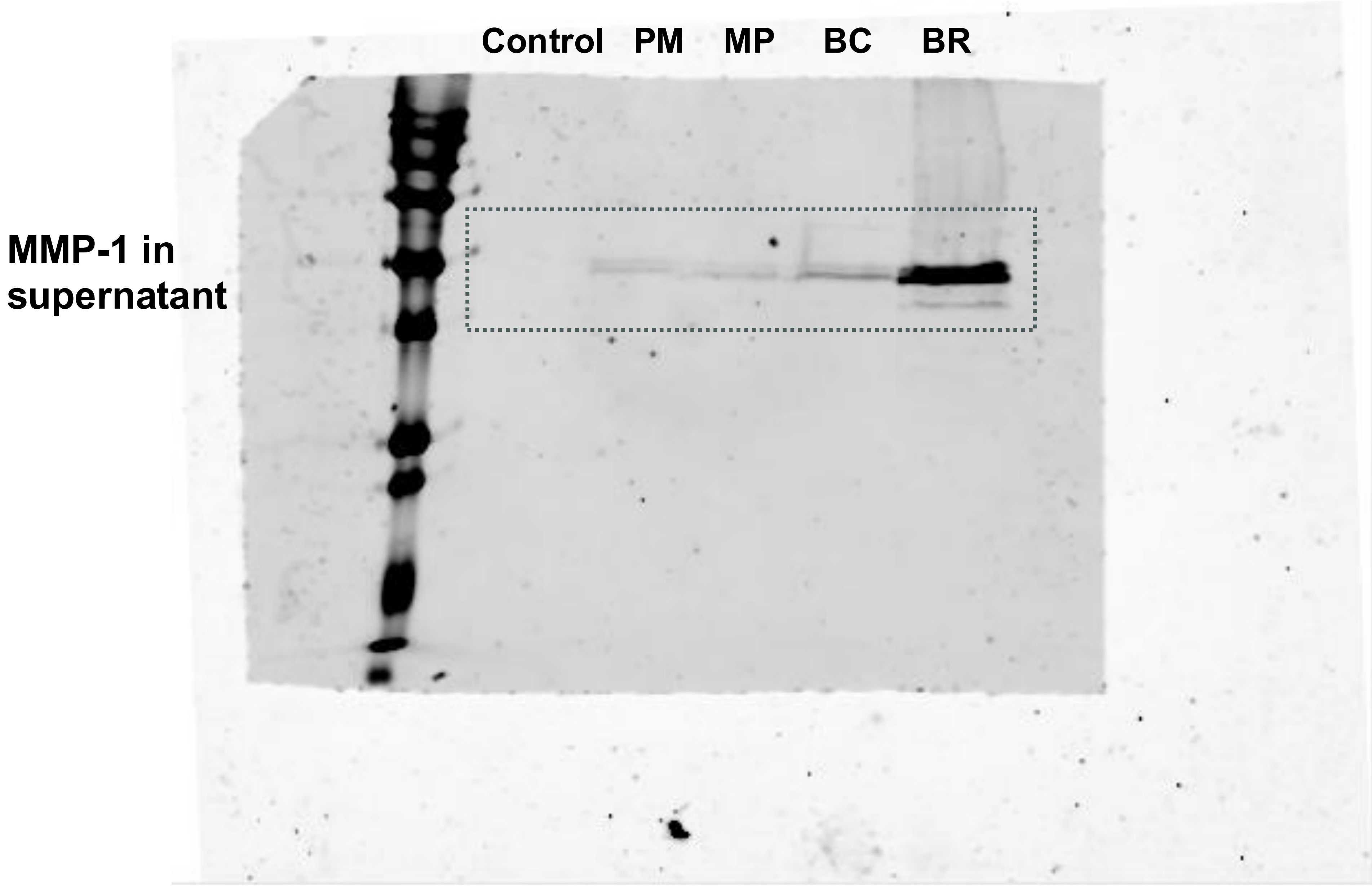

Fig. 4A

intracellular MMP-1

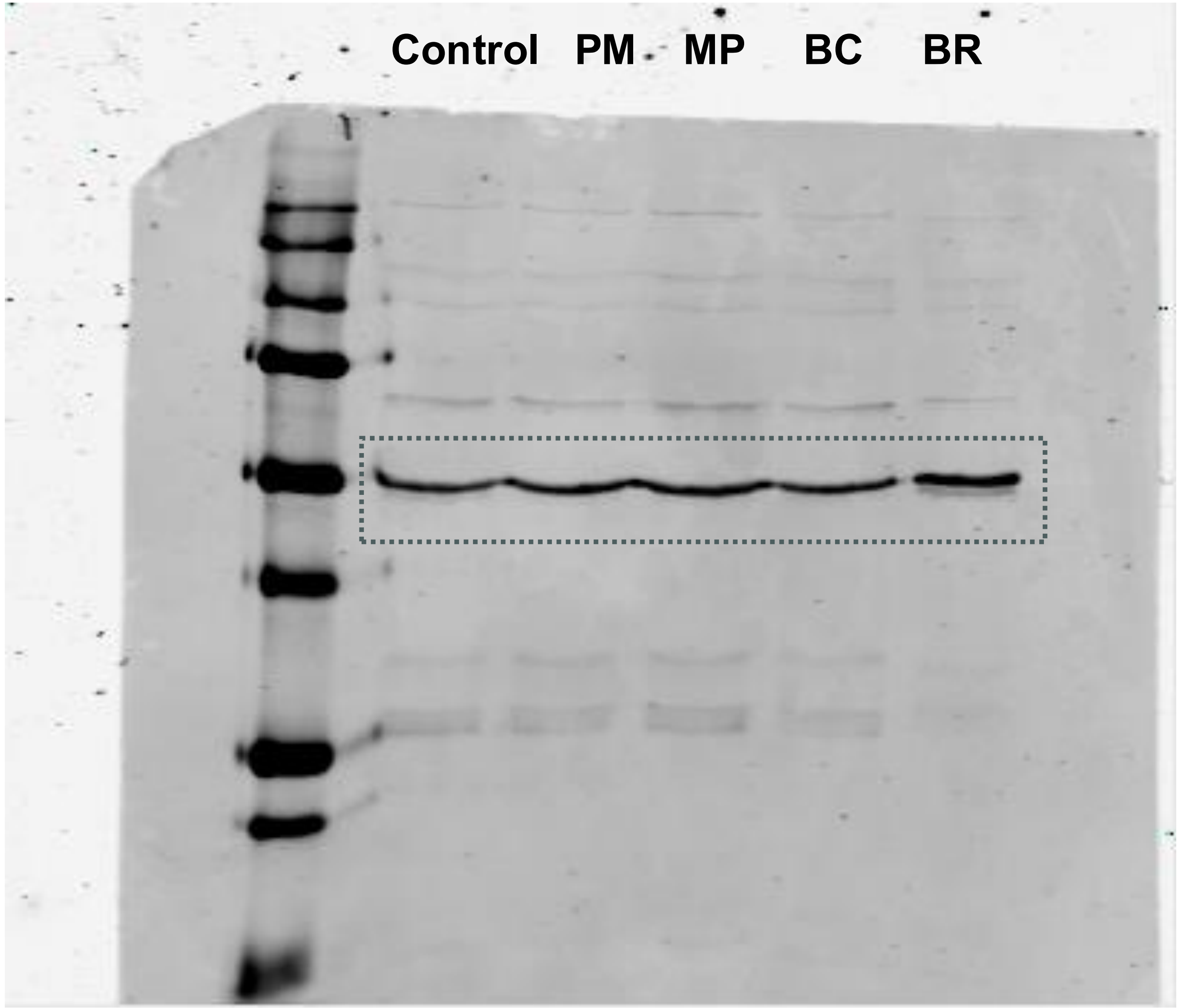

Fig. 4A

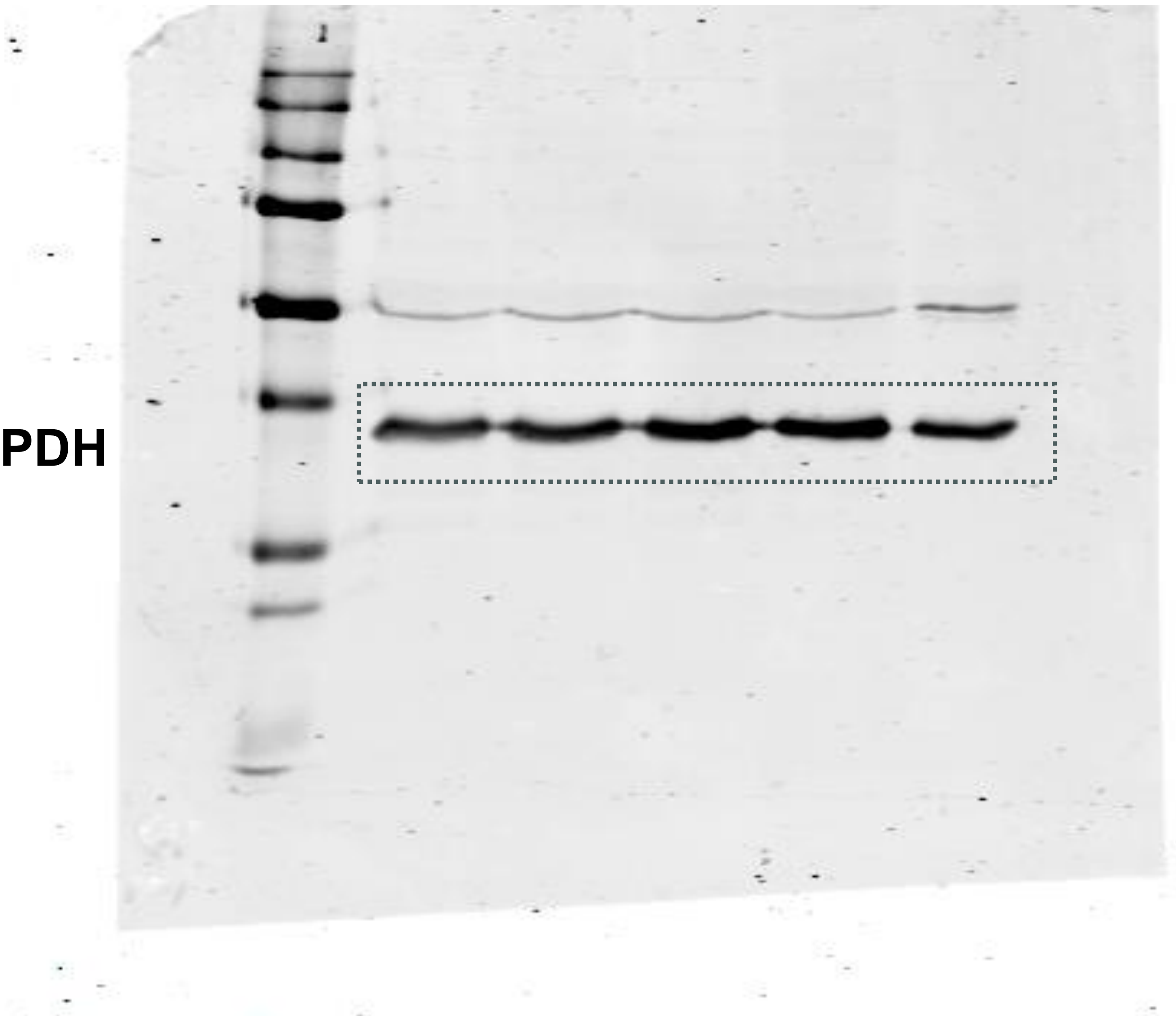

Fig. 5A

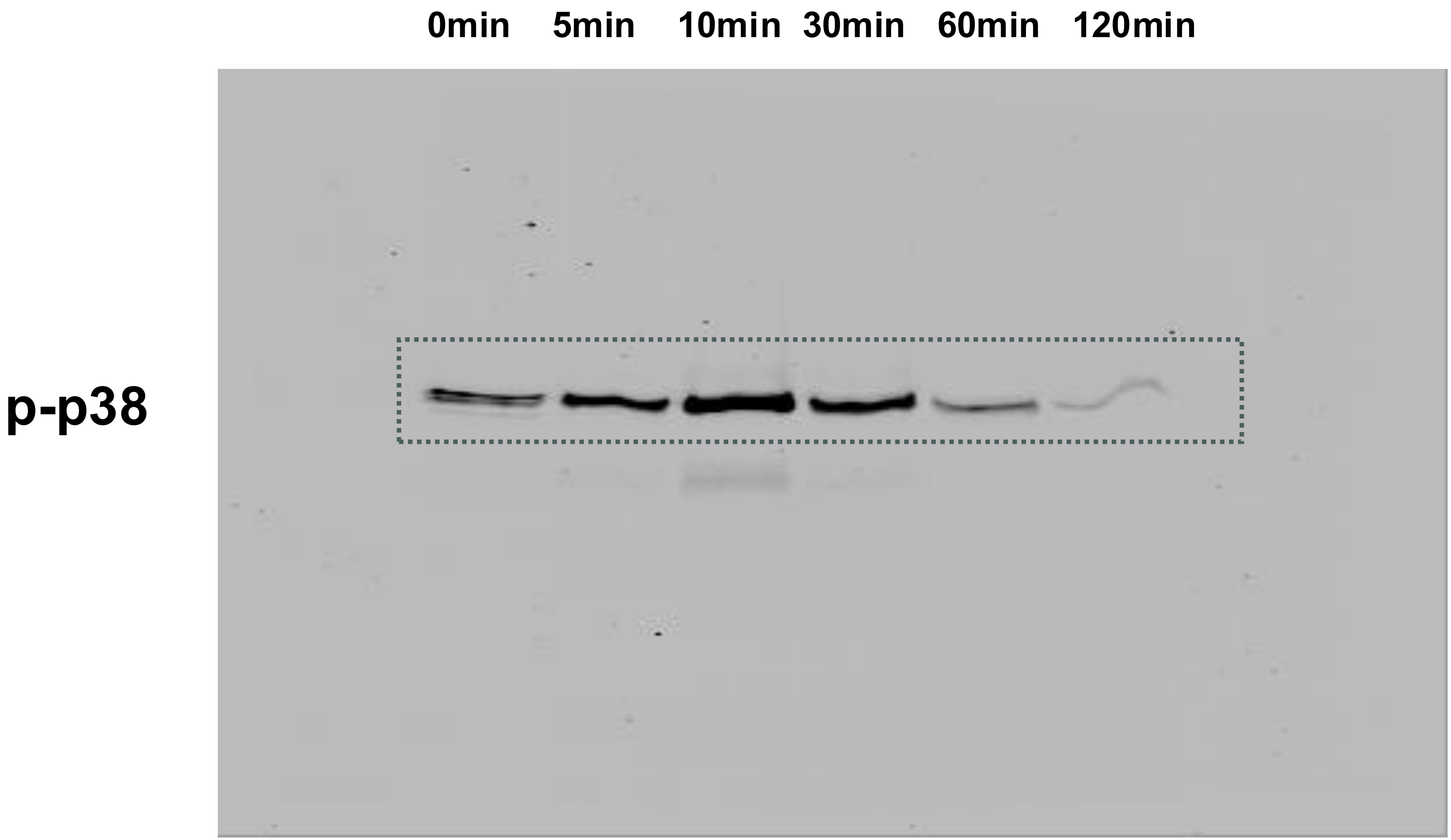

Fig. 5A

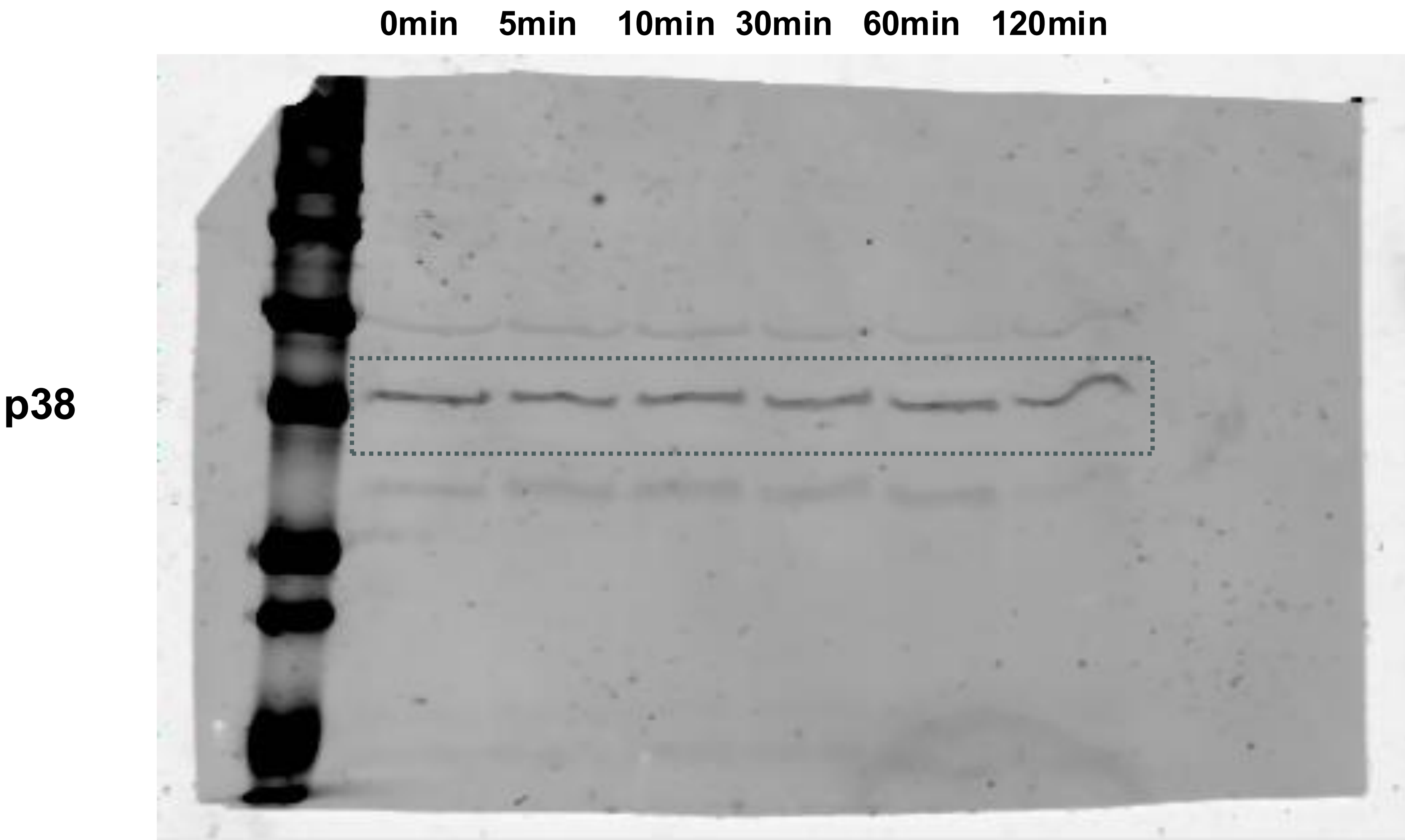

Fig. 5A

p-JNK

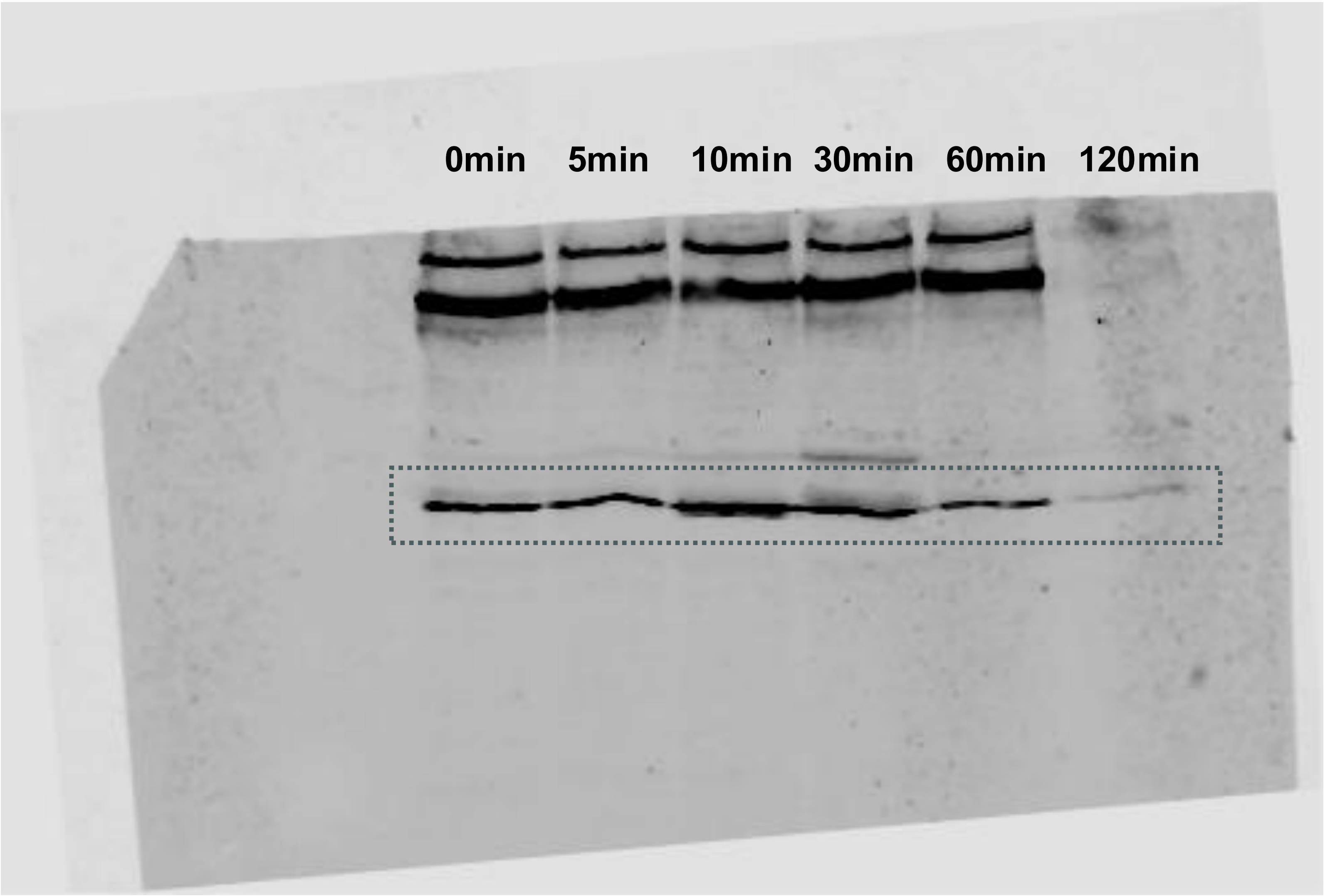

Fig. 5A

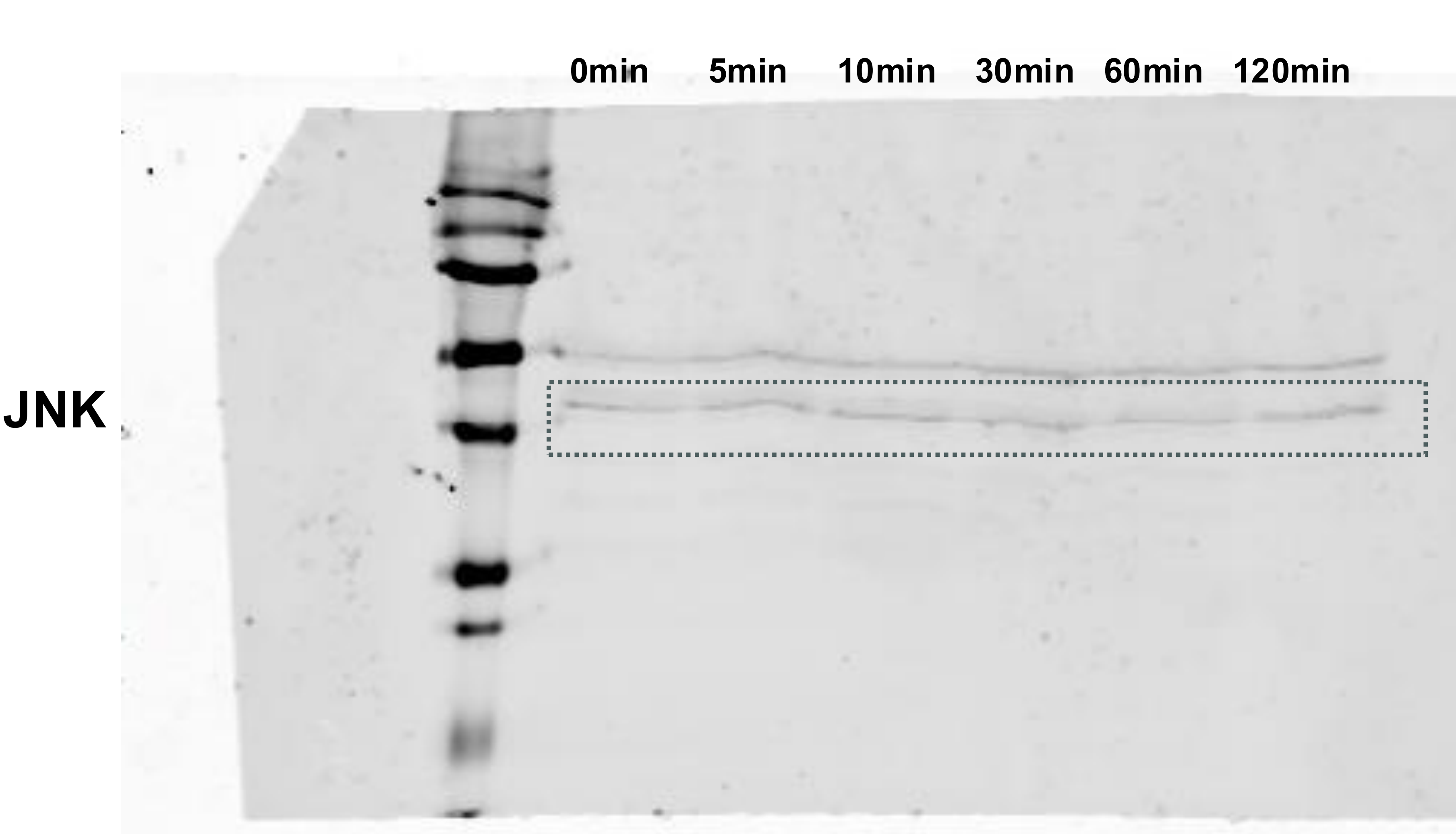

Fig. 5A

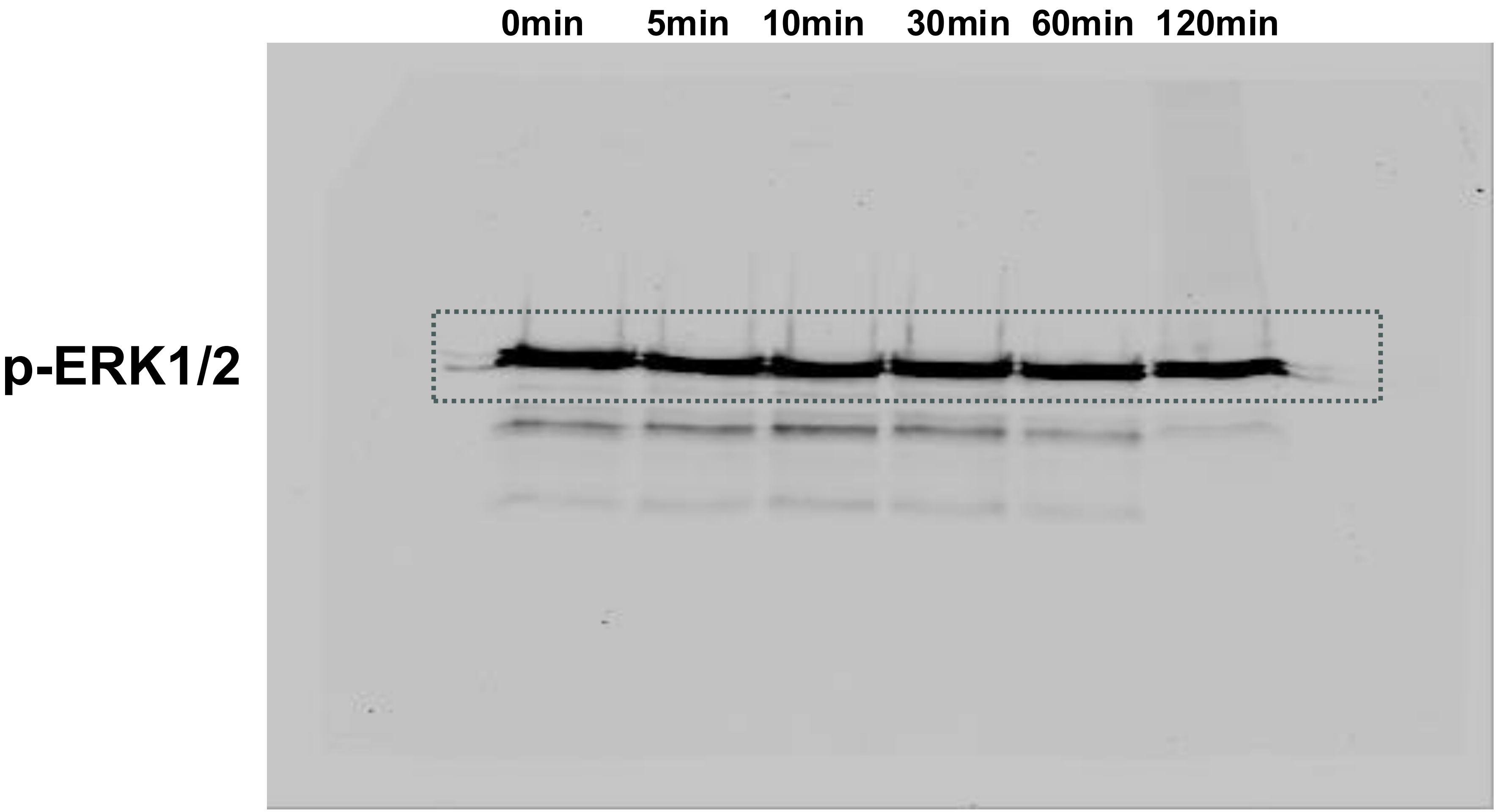

Fig. 5A

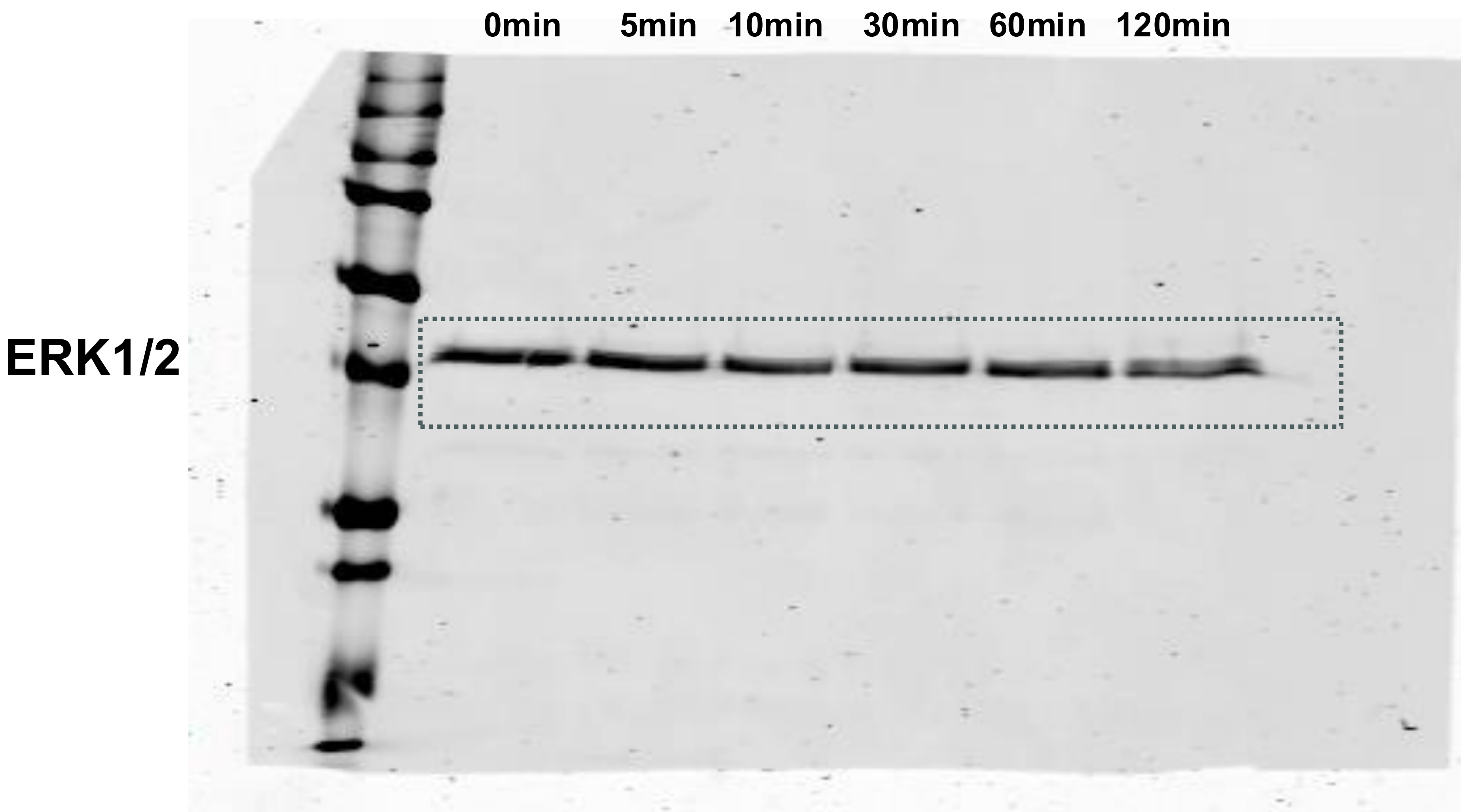

Fig. 5B

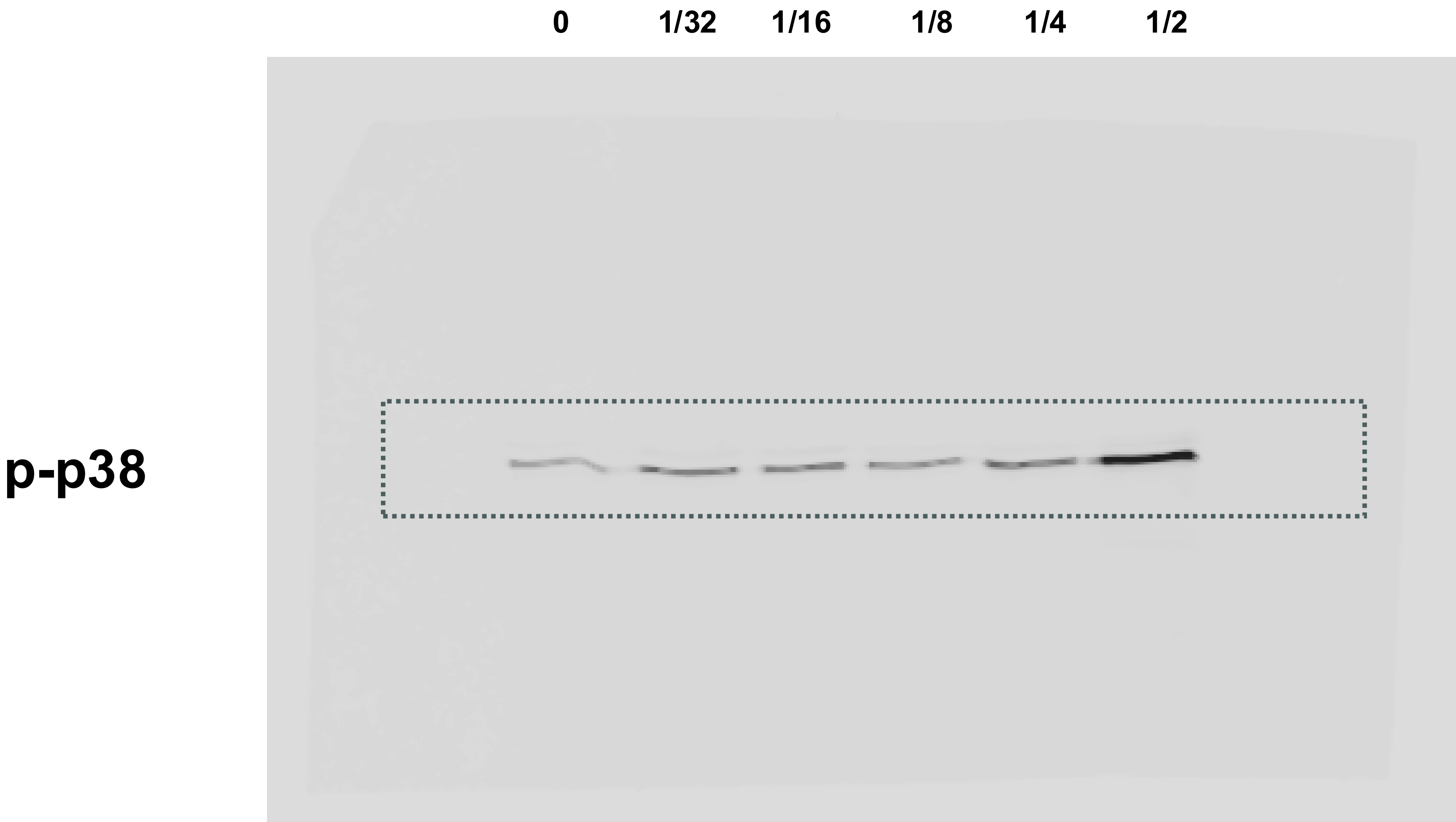

Fig. 5B

p38

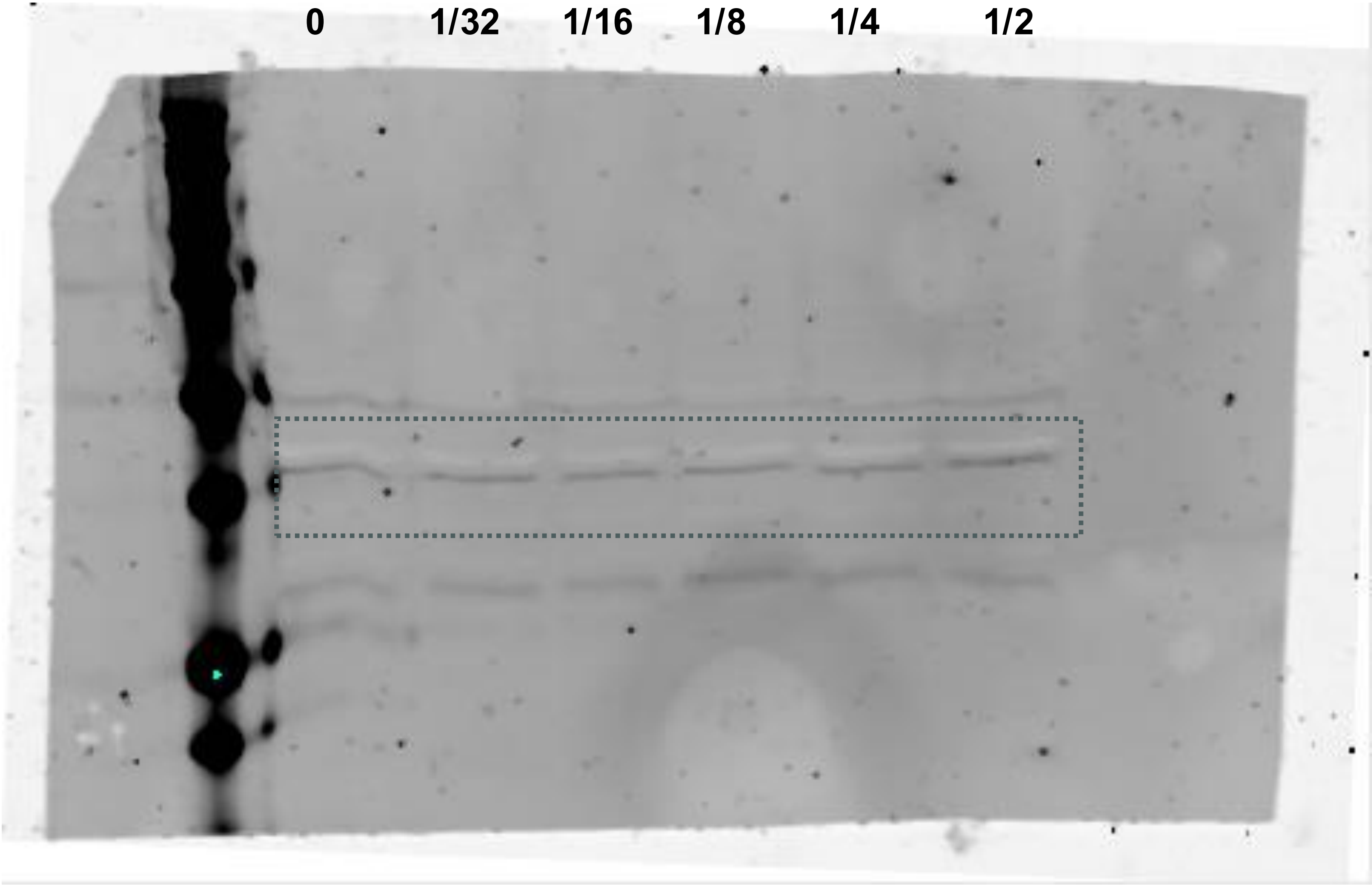

Fig. 5B

p-JNK

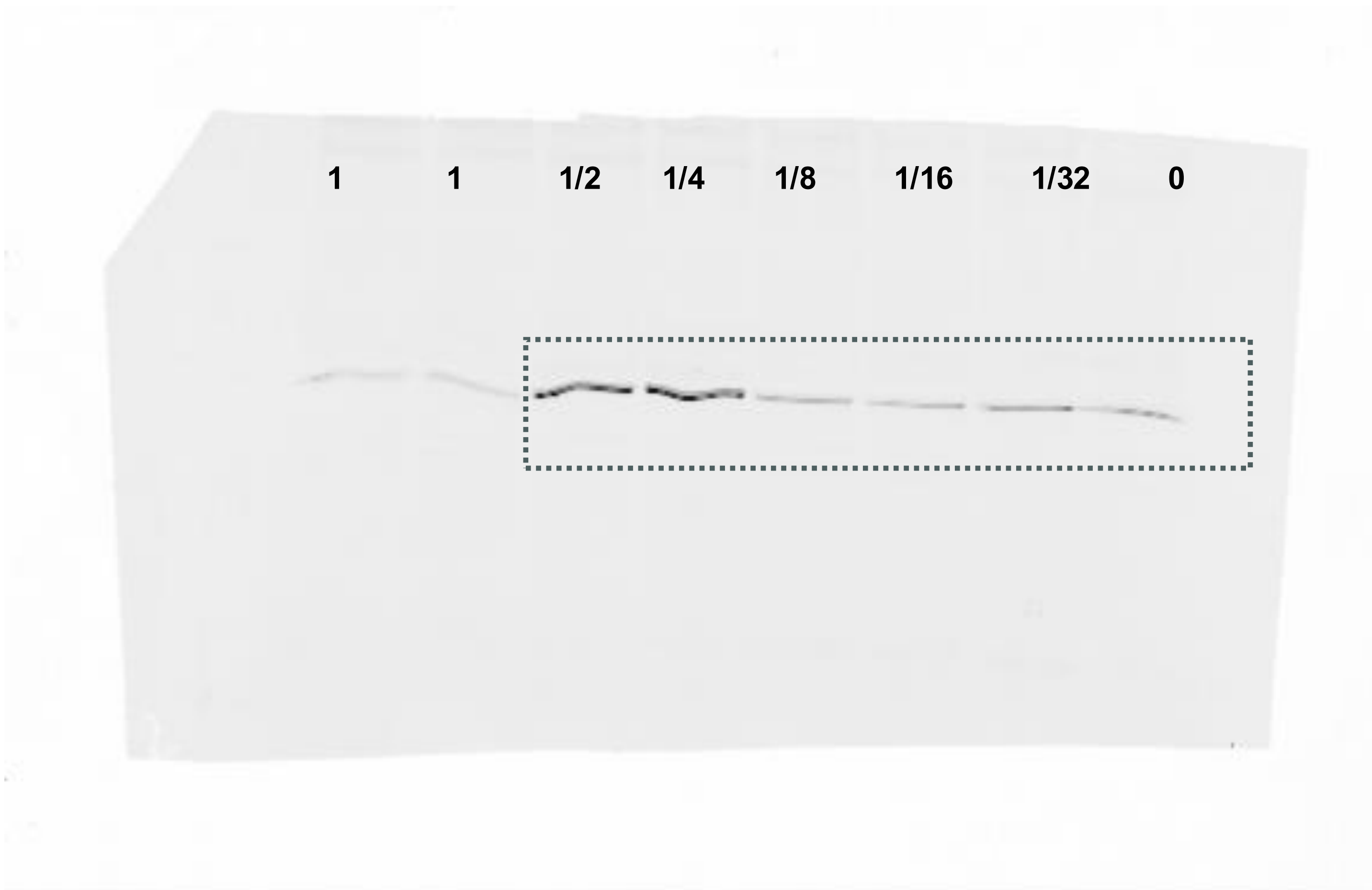

Please note that the sample loading order in the Western blot bands (from ½ to 0) is the reverse of that shown in Figure 5B (from 0 to ½).

Fig. 5B

JNK

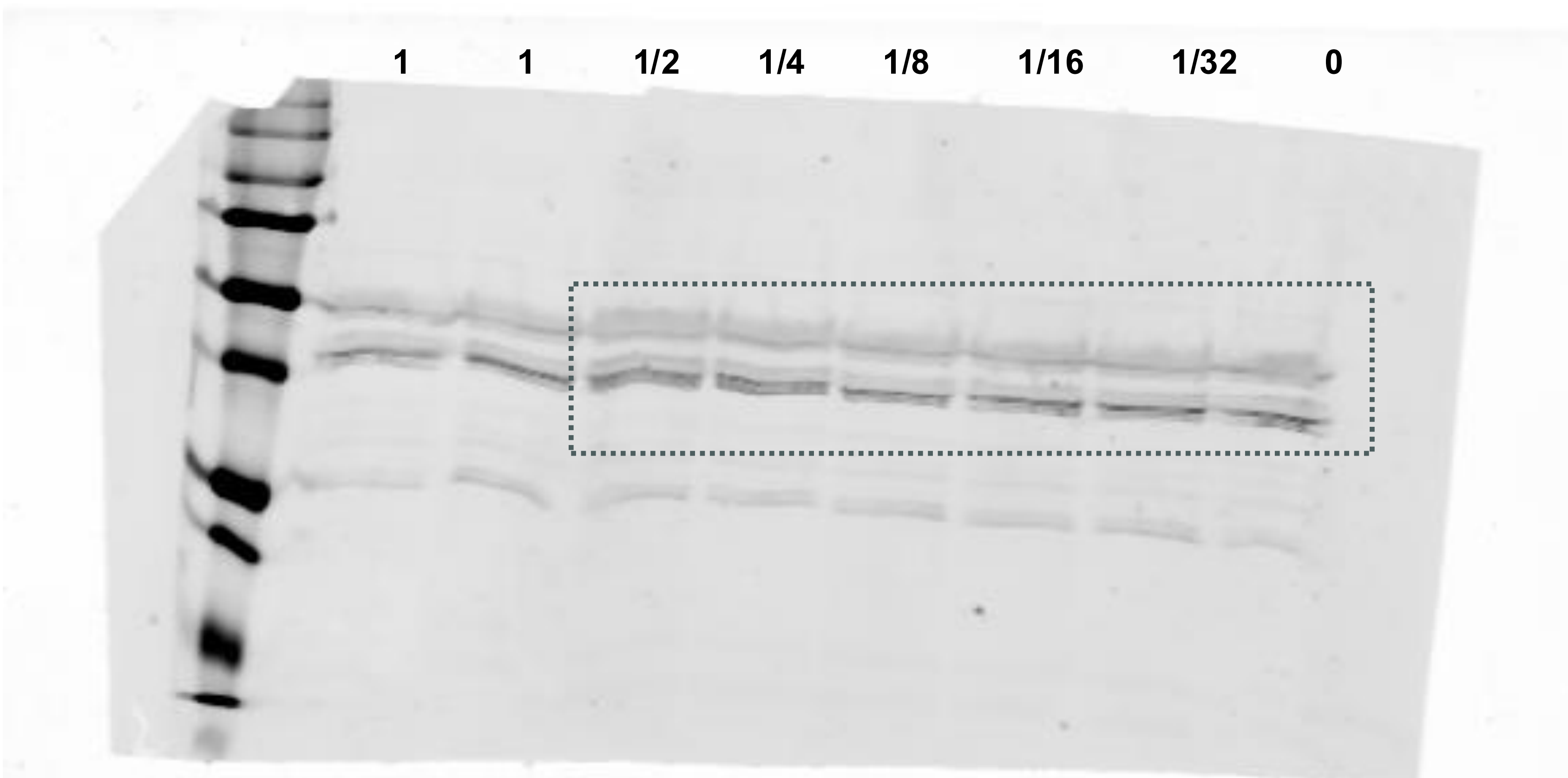

Please note that the sample loading order in the Western blot bands (from ½ to 0) is the reverse of that shown in Figure 5B (from 0 to ½).

Fig. 5B

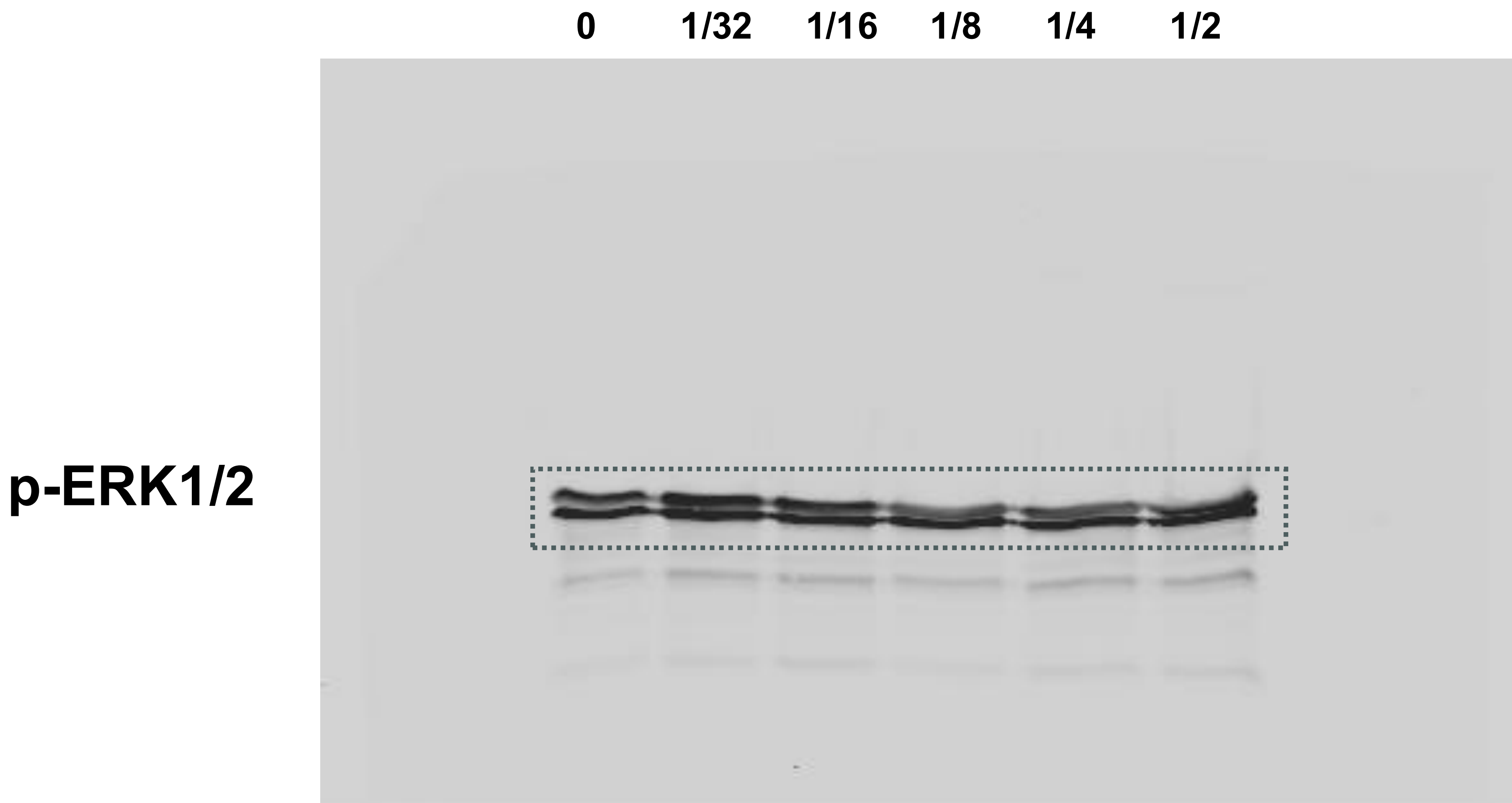

Fig. 5B

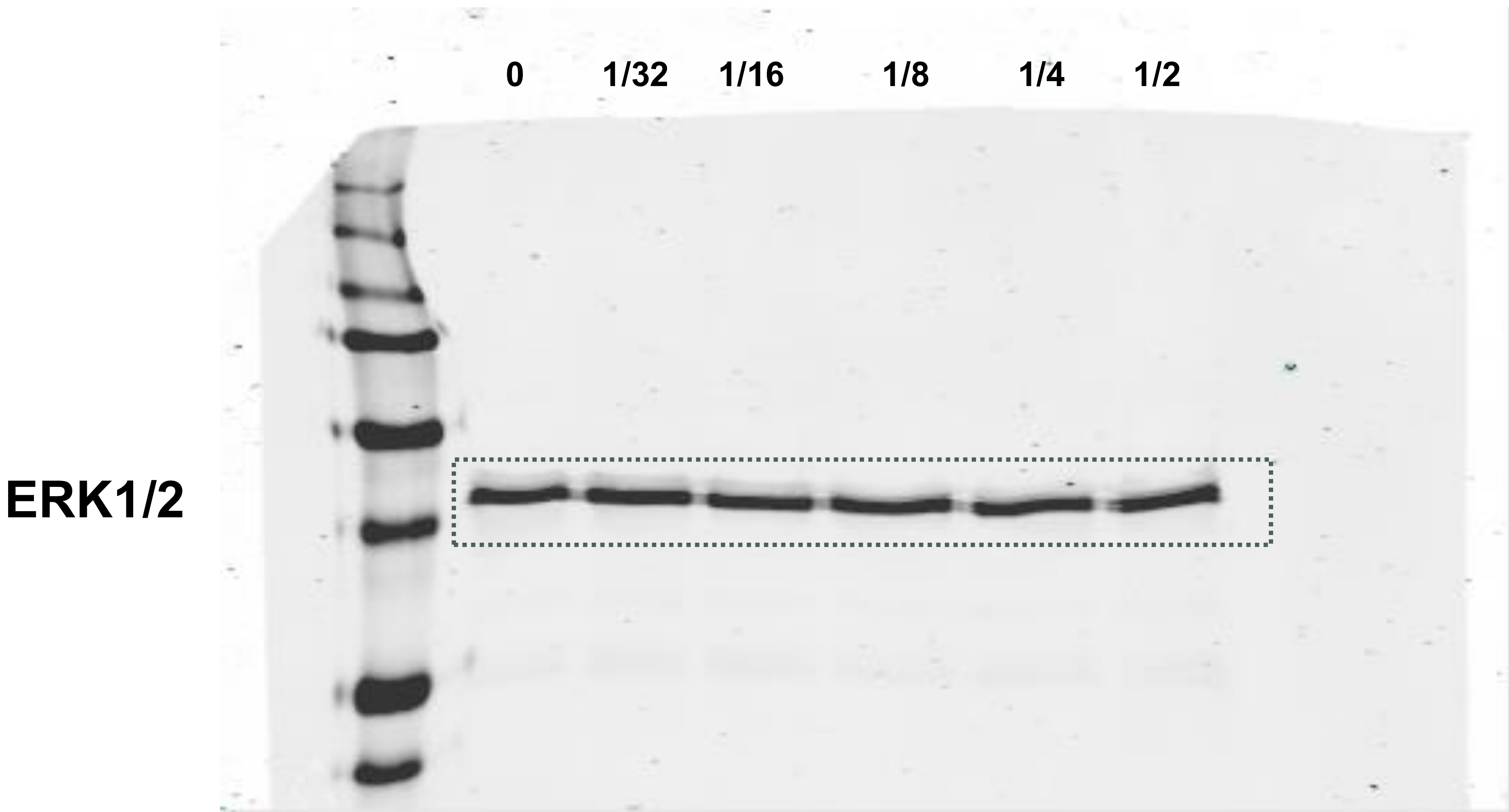

Fig. 6A

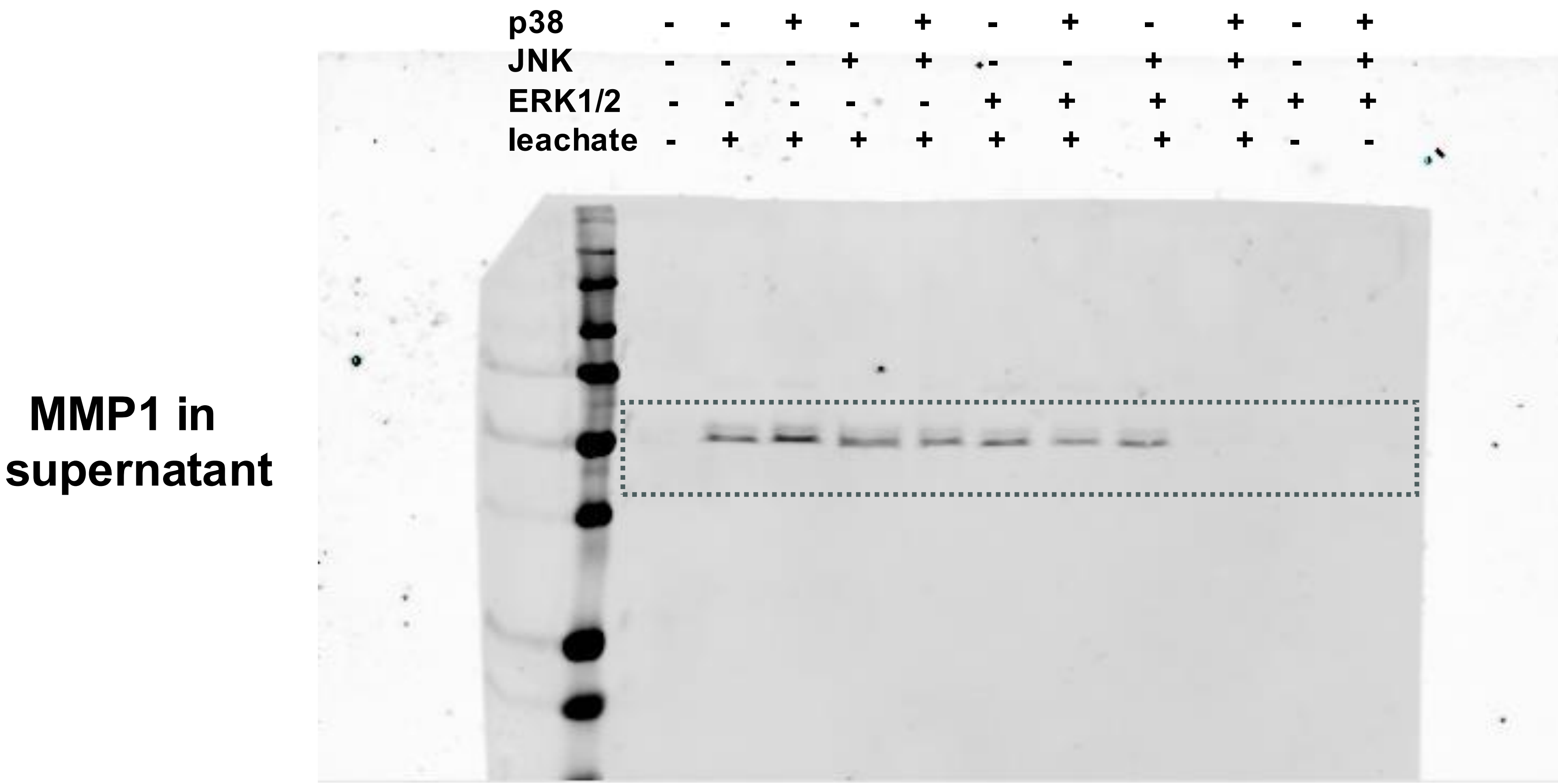

Fig. 6A

p38	-	-	+	-	+	-	+	-	+	-	+
JNK	-	-	-	+	+	-	-	+	+	-	+
ERK1/2	-	-	-	-	-	+	+	+	+	+	+
leachate	-	+	+	+	+	+	+	+	+	-	-

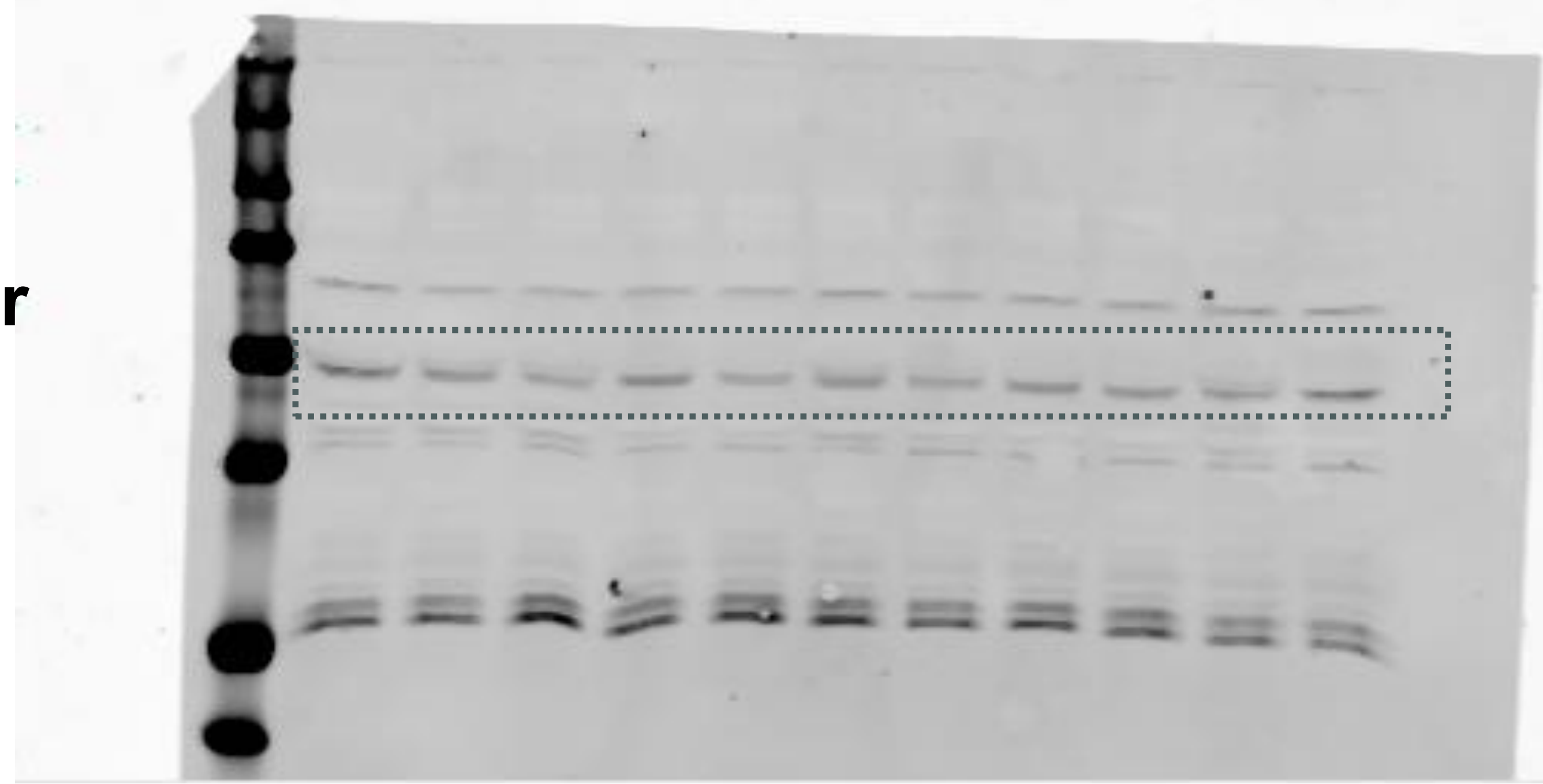

intercellular MMP1

Fig. 6A

p38	-	-	+	-	+	-	+	-	+	-	+
JNK	-	-	-	+	+	-	-	+	+	-	+
ERK1/2	-	-	-	-	-	+	+	+	+	+	+
leachate	-	+	+	+	+	+	+	+	+	-	-

GAPDH

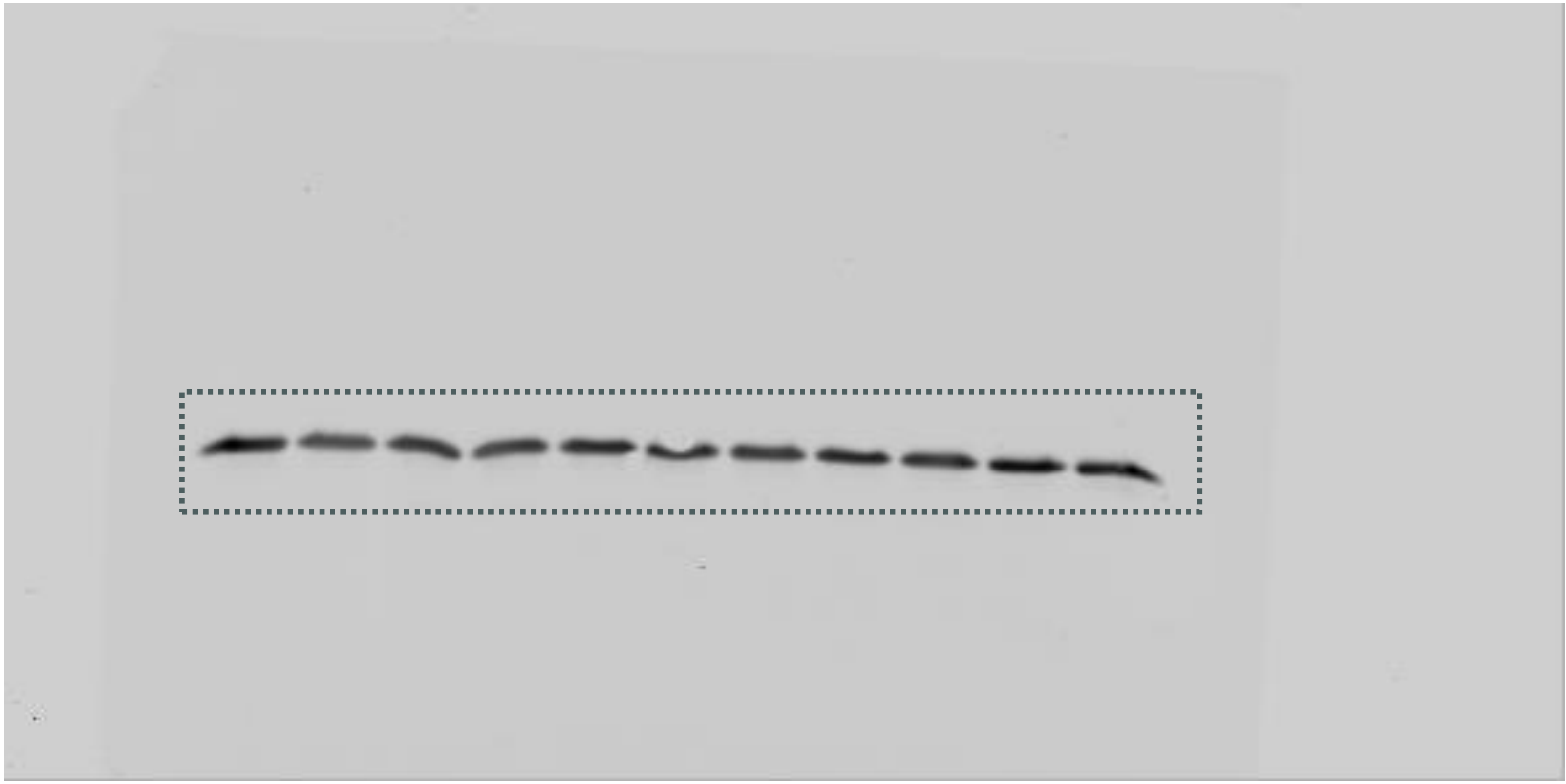

Supplement: Supplementary file 1 [file pone.0359096.s002.pdf]
